# Collective behaviour in 480-million-year-old trilobite arthropods from Morocco

**DOI:** 10.1038/s41598-019-51012-3

**Published:** 2019-10-17

**Authors:** Jean Vannier, Muriel Vidal, Robin Marchant, Khadija El Hariri, Khaoula Kouraiss, Bernard Pittet, Abderrazak El Albani, Arnaud Mazurier, Emmanuel Martin

**Affiliations:** 1 Université de Lyon, Université Lyon 1, ENS de Lyon, CNRS, UMR 5276 Laboratoire de géologie de Lyon: Terre, Planètes, Environnement, Bâtiment Géode; 2, rue Raphaël Dubois, F-69622 Villeurbanne, France; 2 Université de Brest, CNRS, IUEM-UBO, CNRS, UMR 6538 Laboratoire Géosciences Océan, rue Dumont d’Urville, F-29280 Plouzané, France; 3 Musée Cantonal de Géologie, Université de Lausanne, Bâtiment Anthropole, 1015 Lausanne, Switzerland; 4 Université Cadi-Ayyad, Département des Sciences de la Terre, Faculté des Sciences et Techniques, BP 549, 40000 Marrakesh, Morocco; 5 Université de Poitiers, UFR SFA, IC2MP, CNRS, UMR 7285 (HydrASA); 5, rue Albert Turpin, Bâtiment B8, TSA 51106, F-86073 Poitiers, France

**Keywords:** Solid Earth sciences, Palaeontology

## Abstract

Interactions and coordination between conspecific individuals have produced a remarkable variety of collective behaviours. This co-operation occurs in vertebrate and invertebrate animals and is well expressed in the group flight of birds, fish shoals and highly organized activities of social insects. How individuals interact and why they co-operate to constitute group-level patterns has been extensively studied in extant animals through a variety mechanistic, functional and theoretical approaches. Although collective and social behaviour evolved through natural selection over millions of years, its origin and early history has remained largely unknown. *In-situ* monospecific linear clusters of trilobite arthropods from the lower Ordovician (ca 480 Ma) of Morocco are interpreted here as resulting either from a collective behaviour triggered by hydrodynamic cues in which mechanical stimulation detected by motion and touch sensors may have played a major role, or from a possible seasonal reproduction behaviour leading to the migration of sexually mature conspecifics to spawning grounds, possibly driven by chemical attraction (e.g. pheromones). This study confirms that collective behaviour has a very ancient origin and probably developed throughout the Cambrian-Ordovician interval, at the same time as the first animal radiation events.

## Introduction

Modern arthropods provide numerous examples of collective behaviour^1^ and group migrations. The pine processionary caterpillars use pheromone trails and stimuli from abdominal setae to travel head to tail in large groups and over long distances in search of pupation sites (e.g.^2^). Similarly, the non-flying juveniles of the desert locust^3–6^ engage in gregarious behaviour to form huge mobile foraging groups in reaction to a set of mechanical, olfactory and visual stimuli associated with serotonin release^5^. Collective behaviour also occurs in marine crustaceans such as spiny lobsters (*Palinurus)* which perform mass single-file migrations^7–11^ across open substrates either in possible response to storm-induced environmental disturbances, or for reaching spawning grounds (*Palinurus ornatus*^12^). Consistent directional positioning is maintained via tactile contact between followers and/or possible chemical cues^13^. Linear and unidirectional fossil clusters of conspecific trilobite arthropods occur in the Palaeozoic, which have been assumed to result from feeding, reproduction, moulting or sheltering behaviours^14–20^. They have been also assumed to be a solution for reducing hydrodynamic drag effects within the moving groups^21^. Much more enigmatic are the chain-like associations of bivalved euarthropods from the early Cambrian Chengjiang biota, which have been interpreted  to have resulted from collective behaviour and tentatively compared with modern pelagic tunicate chains^22,23^. Some non-linear trilobite clusters are seen as evidence for egg deposition in hatching sites^24^, others as resulting from hypothetical gatherings for protection or moulting^25^. Most of these reported cases of linear or multidirectional clusters lack potentially important constraints on their interpretation such as the sedimentary environment where these animal groups lived and were buried.

Here we describe and analyse quantitatively, numerous linear clusters of *Ampyx priscus* from the Lower Ordovician (upper Tremadocian-Floian, *ca* 480 Ma) Fezouata Shale of Morocco^26–29^, and show that these alignments of trilobites do not result from passive transportation and accumulation by currents but from a collective behaviour. *Ampyx priscus* was probably migrating in groups and used its long projecting spines to maintain a single-row formation by physical contacts possibly associated with mechano-receptors and/or chemical communication. This group behaviour may have been a response to environmental stress due to periodic storms shown by sedimentological evidence, or was associated with reproduction. This record of linear clustering in early euarthropods suggests that intraspecific group-level patterns comparable to those of modern animals already existed 480 million years ago in the early stages of the Great Ordovician Biodiversification Event^30^.

## Results

**Figure 1:**
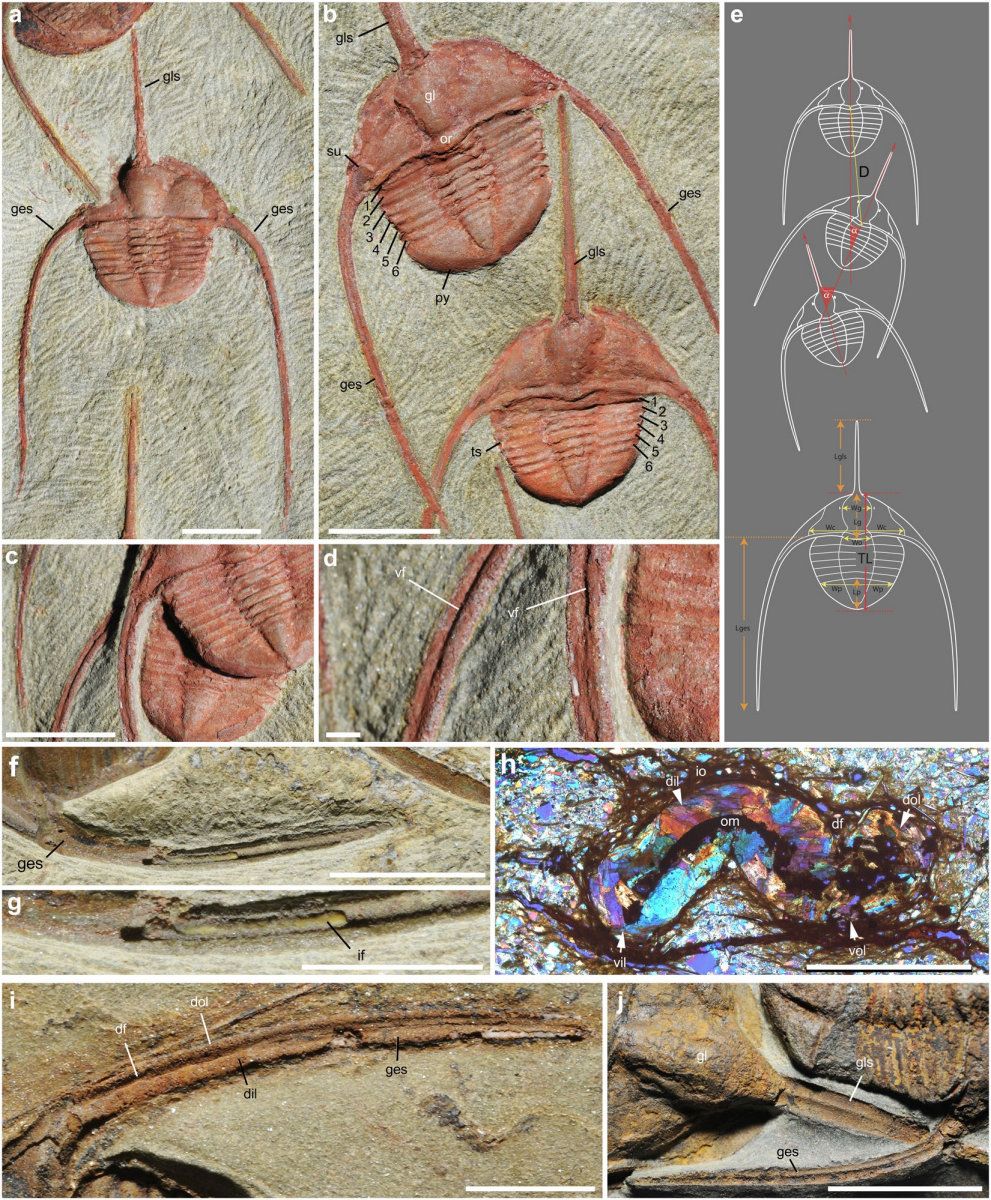
General morphology and parameters of the raphiophorid trilobite *Ampyx priscus* Thoral, 1935, from the Lower Ordovician (Upper Tremadocian-Floian) Fezouata Shale of Morocco (Zagora area). (**a–d**) BOM 2481, overall morphology and details of genal spines. (**e**) Parameters used in measurements. (**f**,**g**) MGL 096718, genal spine showing internal mineralized infilling. (**h**) AA.OBZ2.OI.1, transverse thin section through right genal spine (see general view in Supplementary Fig. 8d). (**i**) MGL 096727, genal spine. (**j**) ROMIP 57013, external mould of glabellar and genal spine showing longitudinal ridge. **a–d**,**f**,**g**,**i**,**j** are light photographs. Abbreviations are as follows: α, angle between the longitudinal axis of two successive individuals; D, distance between two successive individuals in clusters (joins central part of occipital rings); df, dorsal furrow; dil, dorsal inner lobe; dol, dorsal outer lobe; ges, genal spine; gl, glabella; gls, glabellar spine; if, mineralized infilling; io, iron oxide; Lg, length of glabella; Lges, length of genal spine; Lgls, length of glabellar spine; Lp, length of pygidium; om, organic matter; py, pygidium; su, suture; TL, total length; vf, ventral furrow; vil, ventral inner lobe; vol, ventral outer lobe; Wc, width of cranidium; Wg, width of glabella; Wo, width of occipital ring; Wp, width of pygidium; 1–6, 1st to 6th thoracic segment. Scale bars: 1 cm in a–c, f, i, j; 5 mm in  g,h; 1 mm in d.

**Figure 2:**
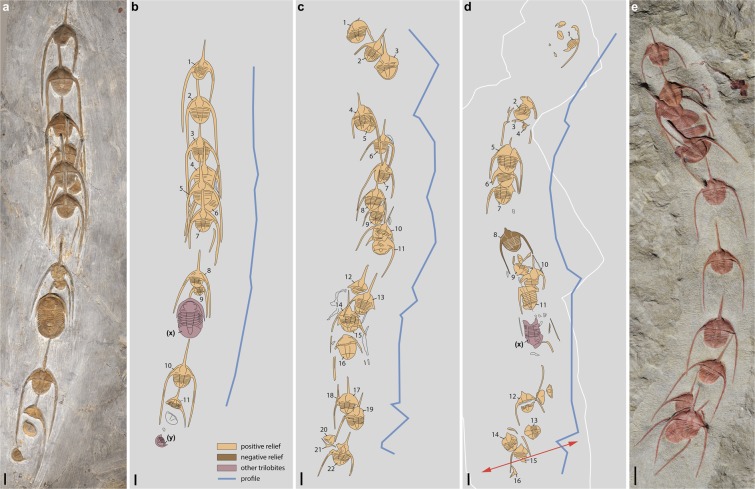
Linear clusters of the raphiophorid trilobite *Ampyx priscus* Thoral, 1935^31^, from the Lower Ordovician (upper Tremadocian-Floian) Fezouata Shale of Morocco (Zagora area). (**a**,**b**) AA.TER.OI.12 (see Supplementary Fig. 2a). (**c**) MGL 096727 (see Supplementary Fig. 5a). (**d**) AA.TER.OI.13 (see Supplementary Fig. 2b). (**e**) BOM 2461 (see Supplementary Fig. 2f). (**a**,**e**) are light photographs. Line drawings from photographs. Segmented blue lines in (**b**–**d**) join the central part of occipital rings of trilobites. Red arrows indicate the position of polished section in Fig. 3. Abbreviations are as follows: (x), *Asaphellus* aff. *jujuanus* (asaphid trilobite); (y), juvenile asaphid trilobite. Scale bars: 1 cm.

The trilobite clusters described here from the upper Tremadocian Fezouata Shale (near Zagora, Morocco) are overwhelmingly dominated by *Ampyx priscus*^31,32^ with rare occurrences of asaphids and calymenids (Fig. 1, Table 1; Supplementary Figs 1–5, Supplementary Tables 1–3) associated with them. *Ampyx priscus* Thoral, 1935^31^ is a raphiophorid trilobite characterized by a stout glabellar spine and a pair of very long librigenal spines projecting posteriorly (Supplementary Text), which occurs at various horizons through the Fezouata Shale (Supplementary Fig. 1a), as isolated individuals or in linear clusters (see also specimens figured by Chatterton and Fortey^16^, plate 1). Trilobites are preserved as internal or external moulds, and are frequently coated with iron oxide. They show no remains of appendages and internal organs. Transverse thin sections (Fig. 1h) indicate that the genal spines had an original tubular structure with a dorsal and ventral furrow and were probably hollow (e.g. infilling; Fig. 1f,g). The glabellar spine is also ventrally striated near its base (Fig. 1j). The distal part of the genal spines runs almost parallel to the longitudinal axis of the animal (Fig. 2a). In each cluster, trilobites are arranged in a linear fashion with their anterior end facing one direction and lie on the surface of a single bedding plane with the dorsal surface of their exoskeleton directed upwards. Only one specimen out of 105 lies upside down (Fig. 2d). The number of *A*. *priscus* specimens in clusters varies from 3 to 22. Clusters with a low number of individuals, may be fragmentary. No specimen is disarticulated (i.e.-cephalon including free cheeks, thoracic segments and pygidium in connection), suggesting that they represent carcasses and not exuviae. *Ampyx* specimens distribute within a relatively narrow size range (more than 75% with a total length (TL) between 16 and 22 mm; Table 1, Supplementary Table 3, Supplementary Fig. 6) and belong to the holaspis stage characterized by a stable number (six) of thoracic segments (Fig. 1b). They probably represent adult or subadult sexually mature animals. Only one juvenile is found in these clusters (see Fig. 2a, most posterior specimen). The angle between the longitudinal axis of two succeeding individuals (α) is usually low and rarely exceeds 45° (see α mean; Table 1, Supplementary Table 3), and the rotation angles within each cluster are randomly clockwise or anticlockwise. This contributes to forming an overall, relatively straight queue with minor local irregularities (Fig. 2). Trilobites facing a direction opposite (α > 90°) to that of their preceding associates are extremely rare (ca 2%; see Supplementary Table 3). The distance between individuals (D; measured from occipital rings) is relatively short and rarely exceeds twice the body length (TL; D mean < 45 mm; Table 1, Supplementary Table 3) giving the trilobite clusters a cohesive appearance. Succeeding specimens are frequently in contact with each other via their long glabellar and genal spines. The length of the glabellar spine (LglS) slightly exceeds TL, that of the genal spines (Lges) is at least twice as long as TL. Overlapping individuals are frequent (Fig. 2). Comparable monospecific linear clusters dominated by *Ampyx priscus* also occur in the upper Tremadocian of the Montagne-Noire, (Hérault, southern France) and share important features with their Moroccan counterparts, such as the anterior polarity, a relatively short inter-individual distance and low angle (α) variations (Supplementary Fig. 7).

**Table 1:** Summary table of measurements of *Ampyx priscus* linear clusters from the Lower Ordovician (Upper Tremadocian-Floian) Fezouata Shale of Morocco (Zagora area).

Clusters	N	TL max	TL min	TL mean	α max	α min	α mean	D max	D min	D mean
LC01	11	19.1	14.9	**16.5**	34.6	**0**.**1**	15.8	43.4	1.7	**24**.**2**
LC02	4	14.7	14.2	**14.4**	15.8	**11**.**1**	11.9	36.7	14.8	**24**.**9**
LC03	9	26.0	20.1	**21.9**	21.5	**1**.**5**	8.8	43.5	17.8	**32**.**5**
LC04	8	19.6	14.9	**17.5**	12.0	**3**.**7**	7.4	71.3	8.6	**33**.**0**
LC06	5	18.7	13.0	**16.8**	14.6	**1**.**0**	14.0	65.2	36.3	**44**.**8**
LC09	3	22.6	19.4	**21.0**	48.6	**32**.**9**	40.7	31.6	30.7	**31**.**1**
LC15	16	23.8	14.1	nd	152.3	**0**	53.8	90.0	7.6	**27**.**4**
LC17	5	20.6	16.8	**18.6**	38.7	**0**.**7**	15.3	23.3	9.4	**19**.**4**
LC18	22	20.6	15.6	**18.3**	60.7	**1**.**5**	17.3	46.1	2.5	**18**.**8**
LC19	6	20.2	18.9	**19.9**	26.7	**3**.**6**	13.5	29.8	10.0	**16**.**2**
LC20	5	21.4	17.6	**19.9**	13.8	**0**.**2**	6.4	48.7	11.8	**25**.**7**
LC21	11	21.1	8.6	**16.5**	15.4	**0**.**2**	7.4	71.8	6.4	**27**.**4**

See Supplementary Tables 1 and 3. Abbreviations are as follows: α, inter-individual angle; D, inter-individual distance; LC, linear cluster ; nd, no data; N, number of trilobite (*Ampyx priscus*) specimens; TL, total length.

**Figure 3:**
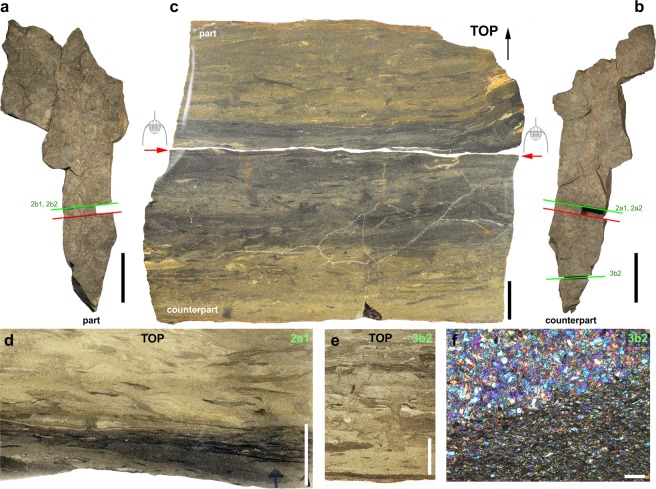
Sediment associated with trilobite clusters. (**a**,**b**) AA.TER.OI.13 (see also Supplementary Figs 8–10), part and counterpart, from the Lower Ordovician (upper Tremadocian-Floian) Fezouata Shale of Morocco (Zagora area). (**c**) Polished section, general view. (**d**,**e**) Thin sections showing details of sedimentary structure. (**f**) Thin section showing grain size and local enrichment in organic matter (lower part). Red arrows indicate bedding plane with *Ampyx* clusters. Location of thin sections indicated by green lines and numbers (see also Supplementary Figs 9–11). Scale bars: 10 cm in a, b; 1 cm in c–e; 50 µm in f.

Polished and thin sections through two rock slabs (Fig. 3; Supplementary Fig. 8) were used to characterize the sedimentary background above and below the trilobite clusters. The sediment is a siltstone evenly composed of well-sorted rounded grains (Fig. 3) of low granulometry (20–30 µm) with a very low percentage of muscovite, clay minerals, and is locally enriched with very fine layers or patches of organic matter (Fig. 3d, Supplementary Figs 8, 9a,b). The original structure of laminae is disturbed by horizontal or subhorizontal bioturbation (Supplementary Fig. 9c,d) which most probably result from the activity of small epibenthic or very shallow endobenthic invertebrates (e.g. euarthropods, worms). No deep burrows or other centimetric sedimentary structures are associated with these trilobite clusters. Bioturbation is indicative of oxic conditions at and slightly below the water-sediment interface. However, its low level of penetration suggests that the redox boundary was probably not deeper than 1.5–2 cm below the sea bed. Elemental mapping (Supplementary Fig. 10) reveals the dominance of Si, Al and K and the ubiquitous presence of Fe (iron oxides and phyllosilicates) and the absence of Ca and S.

## Discussion

### *In situ* preservation

Accumulation layers or shell beds are frequent in the fossil record. They typically consist of disarticulated organisms and heterogenous exoskeletal fragments assembled together and often oriented by currents. The *Ampyx* clusters from the Fezouata Shale have none of these diagnostic features. In contrast, they are made up of articulated monospecific individuals and are not associated with sedimentary structures indicative of sea bottom troughs or burrows. Moreover, the consistent anterior polarity of individuals could hardly be explained by the action of currents. We reject the hypothesis of organisms mechanically accumulated along linear submarine reliefs (e.g. between ripple-marks^14^). Instead, these linear clusters most likely represent *in situ* clusters which retain most of the original position of individuals at the time of their death. This interpretation is strongly supported by geological evidence. Detailed sedimentological analyses^28,29,33,34^ show that the Fezouata Shale is characterized by background sedimentation (mainly argillaceous siltstones) repeatedly disturbed by distal storm sequences made of normally graded, very fine sands and coarse siltstones that locally exhibit small oscillation structures, indicating that the depositional setting was located close to the storm wave base. This facies alternation suggests a depositional setting that lays below the fair-weather wave base and above the storm wave base thus corresponding to marine environments ranging from lower shoreface to lower offshore^28,29,33,34^ (Supplementary Fig. 1b). This style of sedimentary dynamics regulated by storms controlled the settlement of animal communities and also played a major role in the preservation of fossils^33,34^. In the studied area (near Zagora, Morocco) the raphiophorid trilobite biofacies to which *Ampyx priscus* belongs runs slightly above and below the storm wave base (lower part of shoreface to upper part of offshore; see Supplementary Text and Supplementary Fig. 1b).

**Figure 4:**
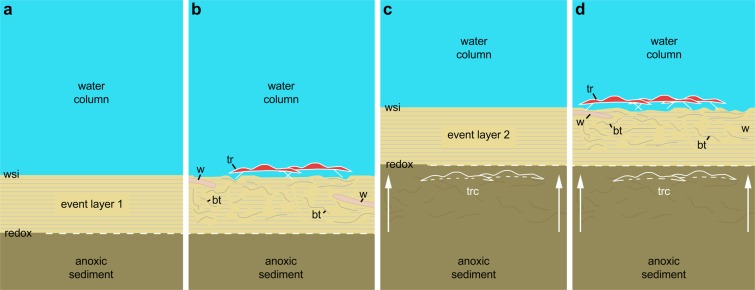
Scenario to explain the *in situ* preservation of the *Ampyx* linear clusters from the Lower Ordovician (Upper Tremadocian-Floian) of Morocco. (**a**) Deposition of a distal tempestite (event layer 1). (**b**) Epibenthic (e.g. trilobites) and shallow endobenthic (e.g. possible worms) organisms settle and generate bioturbation above red-ox boundary. (**c**) Second storm event layer entombs epibenthic fauna *in situ*; red-ox boundary moves upwards (white arrows). (**d**) New faunal recolonization. According to Vaucher *et al*.^34^, distal storm deposits are relatively thin (less than 5 cm) and consist of a waning (base) and waxing (top) phases (subdivision not represented in this diagram), and depositional environment is that of the distal lower shoreface with a possible water depth of approximately 30–70 m. Bioturbation is based on polished and thin sections (Fig. 3 and Supplementary Figs 8 and 9). Abbreviations are as follows: bt, bioturbation; tr, trilobite group (*Ampyx*); trc, trilobite carcasses (*Ampyx*); w, worm; wsi, water-sediment interface.

The amount of sediment deposited during a storm event was probably sufficient to entomb trilobites and other epibenthic animals *in situ* but not powerful enough to take them away. This process is usually invoked to explain the exceptional and *in situ* preservation of marine animals from major Palaeozoic Lagerstätten such as those of the Fezouata Shale^29,33,34^ or the Maotianshan Shale (Chengjiang biota^35^). Hydrogen sulphide released in water by the storm-generated stirring of anoxic sediment has been proposed to explain the sudden death and *in situ* preservation of some Devonian trilobite clusters^21,36^. Similarly, water poisoning may have also participated in rapidly killing *Ampyx* since neither sedimentary disturbances nor body attitudes indicate strong reaction of trilobites to escape burial. Many trilobites (e.g.^37^), including *Ampyx priscus* (Supplementary Fig. 11) had the capacity to enroll as do modern terrestrial isopods when threatened. The extreme rarity of enrolled specimens in linear clusters would support the hypothesis of a very sudden death either induced by water poisoning or by rapid deposition of sediment which hindered enrollment in most specimens. The absence of sulphur in the sediment surrounding trilobite clusters (see elemental mapping; Supplementary Fig. 10) might result from diagenetic processes. The most plausible scenario that led to the preservation of the *Ampyx* clusters from the Fezouata Shale is the following (Fig. 4). Natural linear clusters of trilobites were entombed by sediment generated by distal storms. The upwards migration of the redox boundary created almost immediate lethal conditions around them and maintained carcasses in anoxic conditions, thus limiting scavenging activities and other degradation processes. In summary, we reject the notion that the linear clusters of trilobites result from passive transportation and accumulation by currents and favour a possible behavioural origin.

### Collective behaviour

Various types of trilobite linear clusters have been reported from the Palaeozoic of Morocco^16^, Canada^14^, Poland^20,21^ and other regions (e.g. Portugal^18^), but often lack detailed characterization and key information on their depositional setting. Although some of them tend to be clearly monospecific (e.g. those dominated by *Ampyx* and *Trimerocephalus*^16,21^), others are associated with diverse invertebrates (e.g. bivalves, echinoderms, hyoliths) and contain disarticulated exoskeletons, or consist of randomly oriented individuals. Chatterton *et al.*^14^ and Chatterton and Fortey^16^ interpreted these clusters as possibly resulting from the congregation of trilobites within sub-horizontal burrows made by other animals such as worms but provide no evidence of burrow margins around the trilobite clusters. Our lithological sections (Fig. 3 and Supplementary Figs 8–10) do not reveal any coloured outlines or disturbances in the sediment surrounding trilobites and thus rule out the hypothesis that trilobites may have sheltered collectively in burrows before being trapped and buried *in situ*. Chemical cues or visual attraction to food often drive modern animals to gather and form monospecific clusters of scavengers over carcasses. These aggregations result from individual taxic responses (e.g. myodocope ostracods^38^) and do not strictly correspond to a coordinated group behaviour. The trilobite clusters described here are unlikely to represent such aggregations unless individuals gathered to feed on narrow linear food patches. Moreover, their consistent unidirectional polarity and the lack of associated food remains or traces in sediment make this hypothesis improbable. Another option related to feeding is that *Ampyx* may have fed on suspended particles thus prompting individuals to orientate themselves in the direction of current. If so, we would rather find trilobites lying side by side facing the same direction than aligned in queues.

We propose that the linear clusters formed by *Ampyx priscus* result from the coordinated gathering and locomotion of individuals and therefore suggests a collective and synchronized behaviour. We hypothesize that these trilobites moved in small groups on the seafloor, keeping a single-row formation by physical contacts via their long projecting spines and antennules and/or through chemical communication. Similarly, extant spiny lobsters perform mass single-file migrations by maintaining tactile contact between the tail fan of one individual and the antennular ramus and the tips of the anterior-most walking legs of its follower^7,10^. Such mechanical contacts appear to be essential for group cohesion and for optimal coordinated locomotion. Experimental studies^5^ have shown that the gregarious behaviour of desert locusts was linked to an increase of neurotransmitter (serotonin) driven by mechanical stimuli, visual or olfactory pathways^39–41^. Knowing that *Ampyx priscus* was blind, we hypothesize that mechanosensory stimulation via both genal and glabellar spines, or/and chemical cues, may have been a major trigger that maintained group behaviour. No trace fossils seem to be associated with the trilobite clusters. Possible reasons for this are that either *Ampyx* made no deep imprints into the sediment or that the substrate conditions were unfavourable to preserving traces.

Microscopic observations were unable to locate potential sites (e.g. sensilla pores^42^) of mechano- or chemoreception on the exoskeleton of *A*. *priscus* due to unfavourable conditions of preservation (internal/external moulds with rare cuticular remains). Its remarkably long spiny projections seem to offer optimal conditions for individuals to contact via multiple points (Supplementary Fig. 12) thus increasing the probability for communication via mechanosensory stimulation. This unusual exoskeletal configuration may have facilitated alignment and cohesion within the group. Interestingly, *Ampyxinella* (*Eoampyxinella*) *villebruni*, a raphiophorid trilobite affiliated to *Ampyx* (Supplementary Fig. 13), which lacks a frontal glabellar spine, forms linear clusters characterized by a lower directional consistency compared with those of *Ampyx priscus*. Chemical communication plays a crucial role in the inter-individual relationships of a wide range of extant animals including crustaceans (e.g.^43–45^). Various types of chemosensory sensilla occur over the crustacean body, some of them such as olfactory aesthetascs concentrating on antennules^42^. Such receptors are assumed to be present in Cambrian mandibulates such as *Waptia*^46,47^. Similarly, pores housing a central seta found in Cambrian agnostids^48^ and trilobites (e.g.^49^) may have had a comparable chemosensory function. Chemical communication is often invoked to explain the group behaviour of other trilobites (e.g. blind phacopid *Trimerocephalus*^20,21,50^) and indeed may have played an important role in the collective behaviour of *Ampyx*. However, none of these trilobites provide detailed information on the nature and location of possible receptors.

Fifty-seven percent of the studied clusters from the Fezouata Shale have one and more rarely two non-*Ampyx* trilobite elements which are dominantly complete carapaces of asaphids (mainly *Asaphellus* aff. *jujuanus*), calymenids (*Parabathycheilus* sp.) and dalmanitids; (*Toletanaspis* sp.) (see Supplementary Table 2). Four of these complete trilobites follow the direction of the *Ampyx* cluster in which they seem to be incorporated, whereas the remaining individuals point towards the opposite (2) or intermediate directions (2). In contrast with *Ampyx*, these trilobites had eyes and therefore may have been able to visually detect, track and possibly follow individuals moving in their surroundings. However, the small number of data does not allow for determining whether they may have joined the *Ampyx* groups for some opportunistic reasons or may simply have crossed their migratory path by pure coincidence. As far as we know from the literature^7–11^, no other crustacean species participates in the migration of extant spiny lobsters.

### Triggers and drivers, functions and benefits

#### Spawning congregations and synchronized moulting

Congregation of sexually mature individuals are frequent in extant euarthropods and are often related to reproduction or moulting (e.g.^12,51^). In modern horseshoe crabs, spawning is synchronous with sexually mature individuals gathering along the shore in large numbers for mating (e.g.^52–55^). This behaviour is synchronized through both visual and pheromone communication^56^. *Ampyx* may have performed comparable group migrations to distant spawning grounds during the reproductive season, as proposed by Blazejowki *et al*.^20^ for other trilobites. This hypothesis is supported by the fact that *Ampyx* clusters almost exclusively consist of adult or sub-adult holaspid stages. However, no morphological traits (e.g. body size, spines^57,58^) point to co-existing dimorphs within clusters. Synchronized moulting is frequent in modern crustaceans (e.g. krill^59^) and insects and is assumed to have occurred also in Cambrian euarthropods such as the trilobite *Balcoracania dailyi*^60^ from the lower Cambrian (Series 2, Stage 4) of Australia and *Canadaspis* and *Alalcomenaeus*^61^ from the middle Cambrian Burgess Shale (Series 3, Stage 5) of British Columbia, Canada. This phenomenon brings together a large number of conspecific individuals but does not create any directional polarity among the group (e.g. linear gatherings). Moreover, synchronized moulting, by definition, releases numerous exuviae which are absent from the *Ampyx* clusters.

#### Hydrodynamic cues

Field and laboratory studies on extant spiny lobsters from the Bahamas region have highlighted the possible relation between collective migrations and environmental disturbances^7–11^. A drop in water temperature, higher water turbidity and intense current induced by seasonal storms are assumed to be the main triggers for the mass migrations of these crustaceans, which always take place from highly disturbed shallow coastal areas to the edge of oceanic channels. Most migrants are late juveniles and adults but do not congregate for spawning. The hypothesis that hydrodynamic cues may have driven a comparable behaviour in *Ampyx* is realistic considering that these trilobites were potentially exposed to periodic environmental disturbances generated by storms (see sedimentological evidence above). *Ampyx* is known to occur preferentially across the storm wave base and preferentially in the lower shoreface and upper offshore environments^28,29,62^ (Supplementary Fig. 1b) suggesting possible migrations from storm-influenced to quieter and deeper areas. We hypothesize that its behaviour may have been a collective response to physical stress. Collective migrations of modern crustaceans are assumed to have important advantages over individual ones. Travelling in queues decreases hydrodynamic drag and saves energy (e.g. spiny lobsters^8,10^) and also tends to reduce the probability of detection and attacks from predators by creating confusion in their visual perception (e.g.^63^). *Ampyx* clusters have a relatively small number of individuals and contrast with the long queues seen in spiny lobsters. This might be explained by a natural tendency of trilobites to form smaller groups or by the dispersion of original larger linear clusters by currents. Hydrodynamic signals (direction of storm back current) possibly relayed by sensory setae over the carapace or appendages may also have played an important role in the polarization of locomotion but tactile contacts and/or chemical communication were probably essential to maintaining group cohesion.

**Figure 5:**
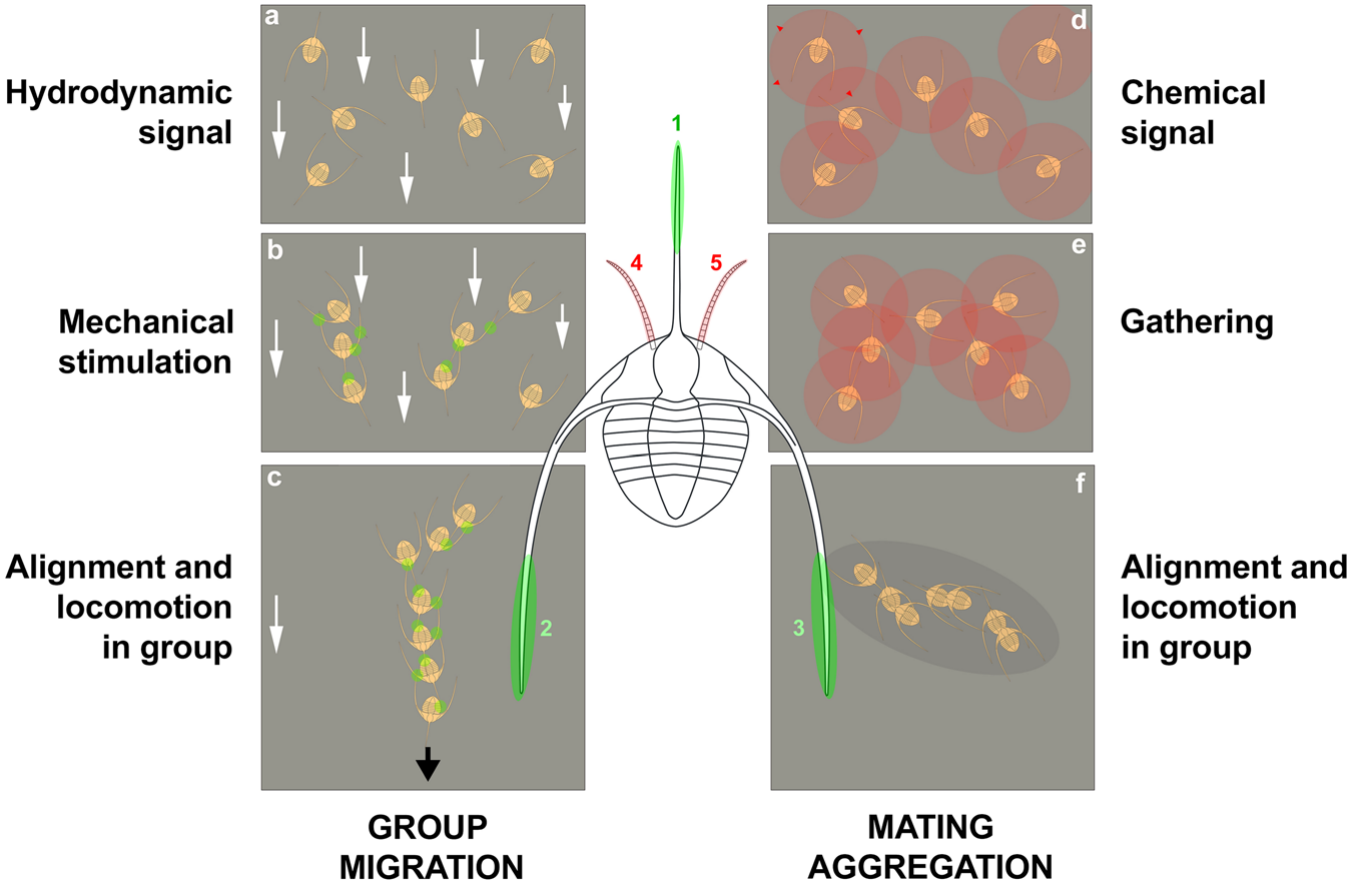
Two non-exclusive hypotheses to explain the linear clusters of *Ampyx priscus* from the Lower Ordovician of Morocco. (**a–c**) Response to oriented environmental stress (e.g. storms); hydrodynamic signal (higher current velocity represented by white arrows) received by motion sensors triggers re-orientation of individuals; mechanical stimulation and/or possible chemical signals cause gathering, alignment and locomotion in group. (**d–f**) Seasonal reproductive behaviour; chemical signals (e.g. pheromones; see red circles and red arrows) cause attraction and gathering of sexually receptive individuals (males and females) and migration to spawning grounds. The alignment of individual may have been controlled by mechanical stimuli (as in **a–c**). Olfactive and mechanical sensors were probably located on the antennules (pink areas 4, 5), and genal and glabellar spines (green areas 1–3), respectively. The exact location of mechanoreceptors is uncertain (possibly on high-relief exoskeletal features such as the glabella).

In summary, two options can be seen as the most likely (Fig. 5): (1) that of a collective behaviour triggered by hydrodynamic cues in which mechanical stimulation detected by motion and touch sensors may have played a major role; (2) that of a possible seasonal reproduction behaviour leading to the migration of sexually mature conspecifics to spawning grounds, possibly driven by chemical attraction (e.g. pheromones). These options are not mutually exclusive. *Ampyx* may have alternatively responded to environmental stress and reproduction signals by adopting the same behaviour.

### Origin of collective behaviour

*Ampyx* shows that collective behaviour in arthropods has a very deep ancestry back to the lower Palaeozoic. This behaviour was necessarily associated with a communication system between individuals involving motion and mechanical sensors, chemical signals and possibly neurotransmitters (e.g. serotonin^5^). Although this behaviour was not mediated by sight because *Ampyx* was blind, it implies neural complexity and the ability to process signals. Numerous arthropods had already evolved sophisticated brains (e.g.^64^), and sensory organs such as antennae and compound eyes (e.g.^47,65–67^) by the Cambrian although their exact functional capabilities often remain conjectural.

*Ampyx* shows how a 480-million-year-old euarthropod may have integrated its neural complexity into a temporary collective behaviour related to seasonal reproduction or triggered by environmental cues. This behaviour is likely to have been widespread among trilobites throughout the Palaeozoic (e.g. Devonian^19^). Another case of enigmatic collective behaviour has been reported in shrimp-like bivalved euarthropods from the early Cambrian Chengjiang biota, which form seemingly closely tied chain-like monospecific associations (*Synophalos*^22,23^, *ca* 520 Ma). These chains strongly recall the migratory queues of spiny lobsters, processionary moth caterpillars or ants but are interpreted^22,23^ as being assembled in the water column and then deposited on the sea bottom through passive sinking. This scenario would suppose an extremely robust interlocking system between individuals and a synchronized group locomotion that have no counterparts in extant arthropods. Males of extant clam shrimps have specialized organs to clasp the edge of the female’s carapace during pairing^68^. However, their behaviour is limited to two sexual partners. Although much information is missing concerning the *Synophalos* clusters (e.g. appendages, relation to sediment, measurements), it appears more likely that, as with *Ampyx*, they represent epibenthic arthropods migrating in groups and buried *in situ* by storm-related or turbiditic sedimentary events. Collective behaviour associated with communication and recognition systems probably evolved through natural selection as the Cambrian radiation proceeded, and developed more extensively during the Great Ordovician Biodiversification Event when ecosystems became increasingly complex. Improving the chances of reproduction and survival to environmental stress are among the advantages that such behaviour may have conferred to euarthropods.

## Methods

Fossils specimens were photographed using a digital camera (D3X-Nikon with Nikon Micro-Nikkor 60 mm lens) and measured (lengths, distances, angles) from high-resolution digital images by using Image J, a public domain processing program. Polished and lithological thin sections were made using standard methods and observed under binocular stereo-microscope (Leica MZ125 and Leica DM750P). Elemental maps were acquired using a Tornado M4 micro-XRF system (Bruker, Germany) equipped with a silicon drift detector and a Rh source operating at 50 kV and 600 µA. A spot size of 40 µm was employed with dwell times of 7 ms/pixel, and mapping was performed under vacuum. Image processing included spectral deconvolution and 3-pixel averaging. Tomographic images of ROMIP 57013 were obtained via the same methods and with the same machine as in Kouraiss *et al*.^69^.

## Supplementary information

SUPPLEMENTARY TEXT

SUPPLEMENTARY TABLES 1-3

SUPPLEMENTARY FIGURES 1-13

## Data Availability

All figured specimens are deposited in fossil collections: Cadi Ayyad University, Faculty of Sciences and Techniques, Marrakesh, Morocco (AA numbers); Musée Cantonal de Géologie, Lausanne, Suisse (MGL); Museum d’Histoire Naturelle de Marseille, France (MHNM); Royal Ontario Museum, Toronto, Canada (ROMIP); Palaeontological Collections, Patrick Bommel, Bizes-Minervois, France (BOM); Palaeontological Collections, Laurent Lacombe, Ouveillan, France (LAC). Correspondence and requests for materials should be addressed to JV.
