# Author Correction: Collective behaviour in 480-million-year-old trilobite arthropods from Morocco

**DOI:** 10.1038/s41598-020-58756-3

**Published:** 2020-01-30

**Authors:** Jean Vannier, Muriel Vidal, Robin Marchant, Khadija El Hariri, Khaoula Kouraiss, Bernard Pittet, Abderrazak El Albani, Arnaud Mazurier, Emmanuel Martin

**Affiliations:** 1 Université de Lyon, Université Lyon 1, ENS de Lyon, CNRS, UMR 5276 Laboratoire de géologie de Lyon: Terre, Planètes, Environnement, Bâtiment Géode; 2, rue Raphaël Dubois, F-69622 Villeurbanne, France; 2 Université de Brest, CNRS, IUEM-UBO, CNRS, UMR 6538 Laboratoire Géosciences Océan, rue Dumont d’Urville, F-29280 Plouzané, France; 3 Musée Cantonal de Géologie, Université de Lausanne, Bâtiment Anthropole, 1015 Lausanne, Switzerland; 4 Université Cadi-Ayyad, Département des Sciences de la Terre, Faculté des Sciences et Techniques, BP 549, 40000 Marrakesh, Morocco; 5 Université de Poitiers, UFR SFA, IC2MP, CNRS, UMR 7285 (HydrASA); 5, rue Albert Turpin, Bâtiment B8, TSA 51106, F-86073 Poitiers, France

Correction to: *Scientific Reports* 10.1038/s41598-019-51012-3, published online 17 October 2019

This Article contains errors in Figure 5: the red circles depicting chemical signals are missing in panel f and the labelling of panels b, c, e and f, as well as the antennule, genal and glabellar spine, are incorrect. As a result, the Figure legend,

**Figure 1:**
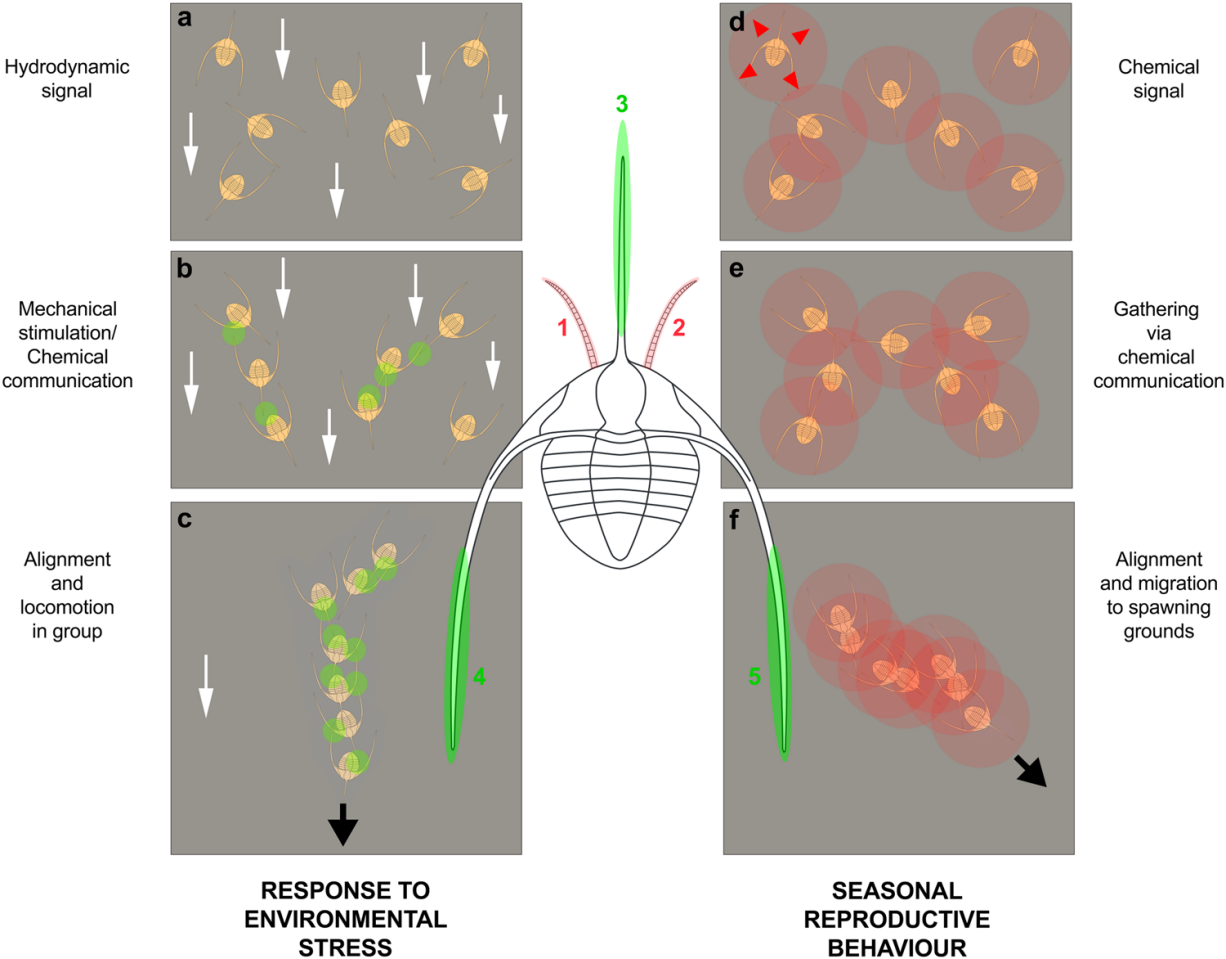
Two non-exclusive hypotheses to explain the linear clusters of *Ampyx priscus* from the Lower Ordovician of Morocco. (**a**–**c**) Response to oriented environmental stress (e.g. storms); hydrodynamic signal (higher current velocity represented by white arrows) received by motion sensors triggers re-orientation of individuals; mechanical stimulation (small green circles) and/or possible chemical signals cause gathering, alignment and locomotion in group. (**d**–**f**) Seasonal reproductive behaviour; chemical signals (e.g. pheromones; see red circles and red arrows) cause attraction and gathering of sexually receptive individuals (males and females) and migration to spawning grounds. The alignment of individual may have been controlled by mechanical stimuli (as in **a**–**c**). Olfactive and mechanical sensors were probably located on the antennules (pink areas 1, 2), and genal and glabellar spines (green areas 3–5), respectively. The exact location of mechanoreceptors is uncertain (possibly on high-relief exoskeletal features such as the glabella).

“(a–c) Response to oriented environmental stress (e.g. storms); hydrodynamic signal (higher current velocity represented by white arrows) received by motion sensors triggers re-orientation of individuals; mechanical stimulation and/or possible chemical signals cause gathering, alignment and locomotion in group. (d–f) Seasonal reproductive behaviour; chemical signals (e.g. pheromones; see red circles and red arrows) cause attraction and gathering of sexually receptive individuals (males and females) and migration to spawning grounds. The alignment of individual may have been controlled by mechanical stimuli (as in a–c). Olfactive and mechanical sensors were probably located on the antennules (pink areas 4, 5), and genal and glabellar spines (green areas 1–3), respectively. The exact location of mechanoreceptors is uncertain (possibly on high-relief exoskeletal features such as the glabella).”

should read:

“(a–c) Response to oriented environmental stress (e.g. storms); hydrodynamic signal (higher current velocity represented by white arrows) received by motion sensors triggers re-orientation of individuals; mechanical stimulation (small green circles) and/or possible chemical signals cause gathering, alignment and locomotion in group. (d–f) Seasonal reproductive behaviour; chemical signals (e.g. pheromones; see red circles and red arrows) cause attraction and gathering of sexually receptive individuals (males and females) and migration to spawning grounds. The alignment of individual may have been controlled by mechanical stimuli (as in a–c). Olfactive and mechanical sensors were probably located on the antennules (pink areas 1, 2), and genal and glabellar spines (green areas 3–5), respectively. The exact location of mechanoreceptors is uncertain (possibly on high-relief exoskeletal features such as the glabella).”

The correct Figure 5 and the correct Figure legend appear below as Figure 1.

