# Supplementary material for: Collective behaviour in 480-million-year-old trilobite arthropods from Morocco
Source: Sci Rep. 2019 Oct 17;9:14941. doi: 10.1038/s41598-019-51012-3 (PMC6797724; doi:10.1038/s41598-019-51012-3)
Supplement: Supplementary file 1 — SUPPLEMENTARY TEXT [file 41598_2019_51012_MOESM1_ESM.pdf]

Collective behaviour in 480-million-year-old trilobite arthropods from Morocco

Jean Vannier, Muriel Vidal, Robin Marchant, Khadija El Hariri, Khaoula Kouraiss, Bernard Pittet, Abderrazak El Albani, Arnaud Mazurier, and Emmanuel Martin

SUPPLEMENTARY TEXT

Supplementary Text | General information on *Ampyx priscus*: morphology and distribution.

Genus *Ampyx* DALMAN, 1827

Type-species : *Ampyx nasutus* Dalman, 1827 (by monotypy)

Ampyx priscus THORAL, 1935

1935. *Ampyx priscus* nov. sp. - Thoral[1], p. 305; Pl. 28, Figs 7-10.
1946. *Ampyx priscus* THORAL - Thoral[2], p. 92.
1966. *Ampyx priscus* THORAL - Dean[3], p. 279; Pl. 3, Figs 1-8; Pl.4, Figs 1-6.
1986. *Ampyx priscus* THORAL - Bérard[4], p. 135; Pl. 12, Figs 1-6, 8, 9.
1998a. *Ampyx* cf. *priscus* THORAL - Vidal[5], p. 61; Pl. 10, Figs 3-9.
2008. *Ampyx* - Chatterton and Fortey[6], p. 76; Pl. 1, Figs 6-9.
2016. *Ampyx priscus* THORAL - Martin et al. [7], p. 144

Description*:* Cephalon with a parabolic anterior outline. Prominent elliptical glabella, strongly convex, raised well above the cheeks, inflated anteriorly, with usually one pair of tiny muscle spots just anterior to the occipital furrow (exceptionally two pairs in rare specimens from Montagne-Noire). These two spots form a constricted area at the base of the glabella. No eyes. Well-developed straight glabellar spine pointing forward, with a tapering shape, slightly tilted upwards, about twice as long as the glabella and probably hollow. Its basal part bears a median ventral groove (Supplementary Fig. 1k). The rest of the spine has an elliptical transverse section. The librigenae are relatively narrow, display a broad sigmoidal boundary with the cranidium, and extend postero-laterally into a pair of long and slender librigenal spines. Distally these elongated spines run almost parallel to the main longitudinal body axis. Their total length is at least twice that of the exoskeleton (excluding glabella spine), exceptionally more than three times. The librigenal spines are grooved on both sides along their entire length. The ventral groove is the deepest and is flanked by an inner and outer lobe. The dorsal groove demarcates a narrow outer lobe from a much broader inner one. Thorax with 6 segments at holaspid stage. Its width reaches a maximum at the second segment, then decreases regularly towards the pygidium. The anterior margin of the pleurae of the first segment is slightly truncated and fits against the posterior margin of the cephalon. Pygidium subparabolic, with a median posterior notch. Its width is about twice its length (sag.). The distal part of the pygidial axis has a triangular shape and does not reach the pygidium posterior margin. The segmentation of the pygidium is very weak on external moulds (anterior pleural furrows curved backwards, only). Internal moulds show up to three rings along the pygidial axis added to the anterior half ring.

Stratigraphic range and palaeogeographic distribution

Ampyx priscus is reported from the Saint-Chinian, La Maurerie and Landeyran formations in the Montagne Noire (Hérault, southern France, about 100 km W of Montpellier[8,9]), dated from the Tremadocian to Floian[10,11]. In Morocco, this species occurs from the late Tremadocian to the Floian of the Fezouata Shale[12] (see Supplementary Fig. 1). In the Meseta (northern part of Morocco) a congeneric species comparable with *A. spongiosus* from Spitzberg was found in possibly younger Floian horizons[13]. In addition to French and Moroccan localities, *A. priscus*

occurs in other localities of the peri-Gonwanan margins such as Sardinia[14] (Pillola, pers. comm. to MV) and possibly Turkey[15, 16] although no precise specific assignment is possible due to the poor preservation of the Turkish material.

Palaeoenvironments

Ampyx belongs to Raphiophoridae, a trilobite family which defines the eponymous biofacies[17,18] in the mid-shelf environments of Avalonia[19]. The raphiophorid biofacies was recognized in the Lower Ordovician of the Anti-Atlas[20] and constrained more precisely by recent sedimentological studies[21,22] in the Zagora area of Morocco. *Ampyx priscus* is typically found associated with with Asaphidae, sometimes with *Parabathycheilus*, *Colpocoryphe* and *Toletanaspis*. In Morocco *A. priscus* occurs in lower shoreface to upper offshore environments and is associated with fine sediments (mainly siltstones, more rarely mudstones) deposited slightly above and below the storm wave base (SWB; see Supplementary Fig. 1) by episodic distal storms (with no reworking of sediment at water-sediment interface) or through background sedimentation[7,12,21,22].

Supplementary references

1. Thoral M. Contribution à l'étude paléontologique de l'Ordovicien inférieur de la Montagne Noire et Révision sommaire de la faune cambrienne de la Montagne Noire, Thèse d'état, Univ. Montpellier, 362 pp. (1935).
2. Thoral M. Cycles géologiques et formations nodulifères de la Montagne Noire. Archives du Museum d'Histoire Naturelle de Lyon 1, 103 pp. (1946).
3. Dean, W. T. The Lower Ordovician stratigraphy and trilobites of the Landeyran valley and the neighbouring district of the Montagne Noire, south-western France. Bulletin of the British Museum (Natural History) Geology 12/6, 245-353 (1966).
4. Bérard, P. Trilobites de l'Ordovicien Inférieur des Monts de Cabrières (Montagne Noire, Hérault, France). Mémoires du Centre de Recherche Géologiques et Hydrologiques de Montpellier 24, 220 pp. (1986).
5. Vidal M. Trilobites (Asaphidae et Raphiophoridae) de l'Ordovicien inférieur de l'Anti-Atlas, Maroc. Palaeontographica A 251, 39-77 (1998a).
6. Chatterton, B. D. E. & Fortey, R. A. in Advances in trilobite research (eds. Rábano, I., Gozalo, R. & García-Bellido, D. 73-79 (Cuadernos del Museo Geominero, nº 9. Instituto Geológico y Minero de España, 2008).
7. Martin, E. L. O. et al. in The Fezouata Biota : an exceptional window on the Cambro-Ordovician faunal transition (eds. Lefebvre, B., Lerosey-Aubril, R., Servais, Th. & Van Roy, P.). 142-154 (Special Issue, vol. 460 Palaeogeography, Palaeoclimatology, Palaeoecology, 2016b).
8. Vizcaino, D. et al. The Lower Ordovician of the southern Montagne Noire. Annales de la Société Géologique du Nord 8, ser.2, 213-220 (2001).
9. Vizcaino, D. and Alvaro, J.J. Adequacy of the Early Ordovician trilobite record in the southern Montagne Noire (France): biases fir biodiversity documentation. Transactions of the Royal Society of Edinburgh, Earth Science, 93, 393-401 (2003).
10. Fortey, R.A. A critical graptolite correlation into the Lower Ordovician of Gondwana. Proceedings of the Yorkshire Geological Society 58, 223-226 (2011).
11. Kröger, B. and Ewans, D.H. Review and palaeoecological analysis of the Tremadocian–early Floian (Early Ordovician) cephalopod fauna of the Montagne Noire, France. Fossil Record 14 (1), 5-34 (2011)
12. Martin, E. L. O. et al. Age and environmental setting of the Lower Ordovician Fezouata Biota (Zagora, Morocco). Gondwana Research 34, 274-283 (2016a).
13. El Hassani A., et al. Découverte d'une macrofaune arénigienne (Ordovicien inférieur) à trilobites et graptolithes dans la région de Rabat, Meseta côtière nord-occidentale (Maroc). Compte-rendu de l'Académie des Sciences, Paris 307, ser.2, 1589-1594 (1988).

14. Pillola, G.L. and Leone F. Lower Ordovician (Arenig) trilobites from SE Sardinia (Italy): palaeobiogeographical and structural implications. Second International Trilobite Conférence, St. Catharines, Ontario p. 43 (1997).
15. Dean, W.T. The Lower Palaeozoic stratigraphy and faunas of the Taurus Mountains near Beysehir Turkey. II. The trilobites of the Seydisehir Formation (Ordovician). Bulletin of the British Museum (Natural History) Geology 20, 3–24 (1971).
16. Dean, W.T. The Lower Palaeozoic stratigraphy and faunas of the Taurus Mountains near Beysehir, Turkey III. The trilobites of the Sobova Formation (Lower Ordovician). Bulletin of the British Museum (Natural History) Geology, 24, 279-348 (1973).
17. Fortey R.A. & Owens R.M. The Arenig series in South Wales: stratigraphy and palaeontology. I. The Arenig series in South Wales. Bulletin of the British Museum (Natural History) Geology 41 (3), 69-307 (1987)
18. Cocks, L.R.M. & Torsvik T.H. European geography in a global context from the Vendian to the end of the Palaeozoic in European Lithosphere Dynamics (eds. Gee, D. G. & Stephenson, R. A.), Geological Society, London, Memoirs, 32, 83–95 (2006).
19. Fortey R.A. and Owens R.M. Early Ordovician (Arenig) stratigraphy and faunas of the Carmarthen disctrict, South-West Wales. Bulletin of the British Museum (Natural History) Geology 30 (3), 225-294 (1978)
20. Vidal M. The trilobite biofacies model: a test in the Early Ordovician of the Anti-Atlas, Morocco. Compte-rendu de l'Académie des Sciences Paris, Earth and Planetary Sciences 327, 327-333 (1998b).
21. Vaucher et al. in The Fezouata Biota: an exceptional window on the Cambro-Ordovician faunal transition (eds. Lefebvre, B., Lerosey-Aubril, R., Servais, Th. & Van Roy, P.). 24-34 (Special Issue, vol. 460 Palaeogeography, Palaeoclimatology, Palaeoecology, 2016).
22. Vaucher R. et al., A wave-dominated, tide modulated model for the Lower Ordovician of the Anti-Atlas, Morocco. Sedimentology 64, 777-807 (2017).
