# Supplementary material for: Collective behaviour in 480-million-year-old trilobite arthropods from Morocco
Source: Sci Rep. 2019 Oct 17;9:14941. doi: 10.1038/s41598-019-51012-3 (PMC6797724; doi:10.1038/s41598-019-51012-3)
Supplement: Supplementary file 2 — SUPPLEMENTARY TABLES 1-3 [file 41598_2019_51012_MOESM2_ESM.pdf]

Collective behaviour in 480-million-year-old trilobite arthropods from Morocco

Jean Vannier, Muriel Vidal, Robin Marchant, Khadija El Hariri, Khaoula Kouraiss, Bernard Pittet, Abderrazak El Albani, Arnaud Mazurier, and Emmanuel Martin

SUPPLEMENTARY TABLES 1-3

Supplementary Table 1| Studied material. Abbreviations are as follows: AA, Cadi Ayyad University, Faculty of Sciences and Techniques, Marrakesh, Morocco (AA= Anti-Atlas); BOM, Palaeontological Collections, Patrick Bommel, Bizes-Minervois, France; LAC, Palaeontological Collections, Laurent Lacombe, Ouveillan, France; MGL, Musée Cantonal de Géologie, Lausanne, Suisse; MHNM, Museum d'Histoire Naturelle de Marseille, France; ROMIP, Royal Ontario Museum, Toronto, Canada (Invertebrate Palaeontology). LC, Linear Cluster.

Supplementary Table 2| Non-Ampyx trilobite elements in linear clusters. LC, Linear Cluster.

Supplementary Table 3| Measurements of *Ampyx priscus* linear clusters from the Lower Ordovician (Upper Tremadocian-Floian) Fezouata Shale of Morocco (Zagora area). α, inter-individual angle; D, inter-individual distance; LC, linear cluster (coll. numbers in brackets; see Supplementary Table 1); Lg, glabella length; Lp, pygidium length; nd, no data; pos/neg, positive/negative relief; TL, total length; Wc, cranidium width; Wg, glabella width; Wo, occipital ring width; Wp, pygidium width; 1 to X, trilobite (*Ampyx priscus*) number. Definition of parameters in Fig. 1e. Framed boxes correspond to maximum and minimum values. Light and dark orange indicate clockwise and anticlockwise rotation, respectively (see α in Fig. 1e).

SUPPLEMENTARY TABLE 1

Cluster N°	COLL N°	Species	Formation	Age	Locality	FIGURES
**LC01**	**BOM 2461**	*Ampyx priscus* Thoral, 1935	Fezouata Shale	Tremadoc	Beni Zoli, 30 km N of Zagora, Morocco	Figs 1a-d, 2e; FigSupFig.2f
**LC02**	**BOM 1442**	*Ampyx priscus* Thoral, 1935	Fezouata Shale	Tremadoc	N of Zagora, Morocco	SupFig. 5e
**LC03**	**BOM 1703**	*Ampyx priscus* Thoral, 1935	Fezouata Shale	Tremadoc	Bou Chrebeb, 28 km NE of Zagora, Morocco	SupFigs 2e, 4a
**LC04**	**BOM 1235**	*Ampyx priscus* Thoral, 1935	Fezouata Shale	Tremadoc	N of Zagora, Morocco	SupFig. 4c
**LC06**	**BOM 1801**	*Ampyx priscus* Thoral, 1935	Fezouata Shale	Tremadoc	N of Zagora, Morocco	SupFig. 4b
**LC09**	**BOM 1704**	*Ampyx priscus* Thoral, 1935	Fezouata Shale	Tremadoc	Bou Chrebeb, 28 km NE of Zagora, Morocco	not figured
**LC15**	**AA.TER.OI.13**	*Ampyx priscus* Thoral, 1935	Fezouata Shale	Tremadoc	Beni Zoli, (ZF5), 30 km N of Zagora, Morocco	Figs 2d, 3; SupFigs 2b 3, 8a-c, 9, 10
**LC16**	**ROMIP 57013**	*Ampyx priscus* Thoral, 1935	Fezouata Shale	Tremadoc	N of Zagora, Morocco	Fig. 1j; SupFig. 11
**LC17**	**MGL 096718**	*Ampyx priscus* Thoral, 1935	Fezouata Shale	Tremadoc	N of Zagora, Morocco	Fig. 1f, g; SupFigs 2c, 5b
**LC18**	**MGL 096727**	*Ampyx priscus* Thoral, 1935	Fezouata Shale	Tremadoc	N of Zagora, Morocco	Fig. 1i, 2c; SupFig. 5a
**LC19**	**MGL 097761**	*Ampyx priscus* Thoral, 1935	Fezouata Shale	Tremadoc	N of Zagora, Morocco	SupFig. 5d
**LC20**	**MHNM 15690-185**	*Ampyx priscus* Thoral, 1935	Fezouata Shale	Tremadoc	N of Zagora, Morocco	SupFigs 2d, 5c
**LC21**	**AA.TER.OI.12**	*Ampyx priscus* Thoral, 1935	Fezouata Shale	Tremadoc	N of Zagora, Morocco	Fig. 2a, b; SupFig. 2a
**LCL22**	**AA.OBZ2.OI.1**	*Ampyx priscus* Thoral, 1935	Fezouata Shale	Tremadoc	Tinzouline (ZF5(2)), N of Zagora, Morocco	SupFig. 8d
**LC23**	**BOM 2480**	*Ampyx priscus* Thoral, 1935	St. Chinian	Tremadoc	Fourbidarias Ravine, St. Chinian, Hérault, France	SupFig. 7a, b
**LC24**	**LAC 001**	*Ampyx priscus* Thoral, 1935	St. Chinian	Tremadoc	Fourbidarias Ravine, St. Chinian, Hérault, France	SupFig. 7c, d
**LC25**	**LAC 002**	*Ampyxinella (Eoampyxinella) villebruni* (Thoral, 1935)	St. Chinian	Tremadoc	Fourbidarias Ravine, St. Chinian, Hérault, France	SupFig. 12a-c
**LC26**	**LAC 003**	*Ampyxinella (Eoampyxinella) villebruni* (Thoral, 1935)	St. Chinian	Tremadoc	Fourbidarias Ravine, St. Chinian, Hérault, France	SupFig. 12d, e

**AA**	Cadi Ayyad University, Faculty of Sciences and Techniques, Marrakesh, Morocco (AA= Anti-Atlas)
**BOM**	Palaeontological Collections, Patrick Bommel, Bizes-Minervois, France
**LAC**	Palaeontological Collections, Laurent Lacombe, Ouveillan, France
**MGL**	Musée Cantonal de Géologie, Lausanne, Suisse
**MHNM**	Museum d'Histoire Naturelle de Marseille, France
**ROMIP**	Royal Ontario Museum, Toronto, Canada (Invertebrate Palaeontology)
**LC**	Linear Cluster

SUPPLEMENTARY TABLE 2

Cluster N°	COLL N°	Non-*Ampyx* trilobite elements	Direction	Figures
**LC01**	**BOM 2461**	disarticulated asaphid (*Asaphellus* aff. *jujuanus*)	opposite direction	SupFig. 2f
LC02	BOM 1442	none		
**LC03**	**BOM 1703**	possibly enrolled asaphid (*Asaphellus* aff. *jujuanus*)	intermediate direction	SupFigs 2e, 4a
		incomplete (pygidium + posterior part of thorax)	opposite direction	SupFig. 4a
LC04	BOM 1235	none		
**LC06**	**BOM 1801**	complete undet. asaphid	opposite direction	SupFig. 4b
		complete dalmanitid (*Toletanaspis* sp.)	opposite direction (not within cluster area)	SupFig. 4b
LC09	BOM 1704	none		
**LC15**	**AA-TER-OI.13**	almost complete asaphid (*Asaphellus* aff. *jujuanus*)	opposite direction	SupFig. 2b
LC16	ROMIP 57013	none		
**LC17**	**MGL 096718**	complete calymenid (*Parabathycheilus* sp.)	same direction	SupFig. 2c
LC18	MGL 096727	none		
**LC19**	**MGL 097761**	almost complete calymenid (*Parabathycheilus* sp.)	same direction	SupFig. 5d
**LC20**	**MHNM 15690-185**	complete asaphid (*Asaphellus* aff. *jujuanus*)	same direction	SupFig. 2d
**LC21**	**AA.TER.OI.12**	complete asaphid (*Asaphellus* aff. *jujuanus*)	same direction	Fig. 1a; SupFig. 2a
		larval undet. asaphid	intermediate direction	Fig. 1a
LC22	AA.OBZ2.OI.1	none		

AA Cadi Ayyad University, Faculty of Sciences and Techniques, Marrakesh, Morocco (AA= Anti-Atlas)
BOM Palaeontological Collections, Patrick Bommel, Bizes-Minervois, France
LAC Palaeontological Collections, Laurent Lacombe, Ouveillan, France
MGL Musée Cantonal de Géologie, Lausanne, Suisse
MHNM Museum d'Histoire Naturelle de Marseille, France
ROMIP Royal Ontario Museum, Toronto, Canada (Invertebrate Palaeontology)

LC Linear Cluster

SUPPLEMENTARY TABLE 3 (part 1/2)

LC01 (BOM 2461) N=11

	1	2	3	4	5	6	7	8	9	10	11
pos/neg	pos	pos	pos	pos	pos	pos	pos	pos	pos	pos	pos
Lg (mm)	5.97	6.13	5.92	5.96	7.07	7.29	8.22	7.54	7.44	8.20	nd
Wg	4.65	4.76	4,75	4.50	4.89	5.89	6.84	5.15	5,68	6.16	nd
Wo	4.20	4.15	4.14	4.87	4.44	4.68	5.42	4.76	4,9	5.39	nd
Wc	13.12	14,79	13,7	14,42	15.78	17.60	18.81	15.06	18,05	19.96	no
Lp	3.37	4.05	4.33	4.36	3.84	2.64	4.48	3.86	nd	4.61	4.90
Wp	10.21	9.92	11.39	14.76	12.05	10.86	14.12	10.72	nd	13.13	10.81
TL	15.20	14.94	16.41	16.28	16.14	15.62	19.07	16.25	nd	18.41	nd
Lg/Wc	0.45	0.41	0.43	0.41	0.44	0.41	0.43	0.50	0.41	0.41	nd
D (mm)	0	24.63	10.58	27.83	29.54	43.39	42.23	24.35	25.78	12.05	1.67
D/meanTL		1.49	0.64	1.69	1.79	2.63	2.56	1.48	1.56	0.84	0.20
α (°)	0	7.65	26.15	3.07	34.63	4.41	17.59	21.06	13.48	0.13	29.45

mean TL (mm)	16.48
mean α (°)	15.76
mean D (mm)	24.20

LC02 (BOM 1442) N=4

	1	2	3	4
pos/neg	pos	pos	pos	pos
Lg (mm)	5.09	6.03	5.52	5.90
Wg	4.90	5.18	5.23	4.93
Wo	2.45	3.32	3.16	3.54
Wc	13.18	15.54	13.20	16.02
Lp	nd	4.09	3.49	3.83
Wp	7.04	11.49	10.04	9.67
TL	nd	14.24	14.45	14.68
Lg/Wc	0.38	0.39	0.42	0.37
D (mm)	0	14.76	23.25	36.68
D/meanTL		1.02	1.61	2.54
α (°)	0	15.83	8.86	11.13

mean TL (mm)	14.45
mean α (°)	11.94
mean D (mm)	24.90

LC03 (BOM 1703) N=9

	1	2	3	4	5	6	7	8	9
pos/neg	pos	pos	pos	pos	pos	pos	pos	pos	pos
Lg (mm)	9.36	nd	10.44	9.74	8.89	8.26	8.94	10.45	9.11
Wg	6.09	nd	6.957	5.94	5.32	5.65	7.14	6.52	5.95
Wo	4.60	nd	5.954	6.11	4.91	4.84	nd	5.82	5.50
Wc	17.89	nd	22.47	21.29	18.41	19.23	22.13	21.44	19.15
Lp	4.57	nd	6.80	5.01	4.31	5.21	nd	4.21	5.23
Wp	11.30	nd	15.52	13.90	10.80	12.1	nd	12.49	13.26
TL	20.59	nd	25.96	22.60	20.13	20.63	nd	21.85	21.62
Lg/Wc	0.52	nd	0.46	0.48	0.48	0.43	0.40	0.49	0.47
D (mm)	0	nd	31.67	32.31	43.54	38.13	31.9	17.78	31.91
D/meanTL			1.44	1.47	1.99	1.74	1.45	0.81	1.46
α (°)	0	3.51	1.49	7.40	16.41	21.55	7.88	7.68	4.70

mean TL (mm)	21.91
mean α (°)	8.83
mean D (mm)	32.46

LC04 (BOM 1235) N=8

	1	2	3	4	5	6	7	8
pos/neg	pos	pos	pos	pos	pos	pos	pos	pos
Lg (mm)	6.60	5.75	7.26	6.87	7.00	6.69	7.180	8.03
Wg	5.22	4.37	5.50	4.92	4.67	4.94	4.44	4.19
Wo	3.66	nd	4.44	nd	4.66	3.89	3.86	nd
Wc	16.20	13.38	17.66	13.92	15.23	17.45	14.82	16.09
Lp	3.66	nd	4.81	nd	4.41	4.12	3.86	4.03
Wp	10.17	nd	11.89	nd	10.62	10.19	10.00	10.58
TL	16.23	14.88	19.25	nd	17.91	17.53	17.31	19.60
Lg/Wc	0.41	0.43	0.41	0.49	0.46	0.38	0.48	0.50
D (mm)	0	16.60	12.70	31.18	8.58	27.75	71.30	63.29
D/meanTL		0.94	0.72	1.78	0.49	1.58	4.07	3.61
α (°)	0	4.48	12.03	3.73	6.99	8.57	7.33	8.61

mean TL (mm)	17.53
mean α (°)	7.39
mean D (mm)	33.05

LC06 (BOM 1801) N=5

	1	2	3	4	5
pos/neg	pos	pos	pos	pos	pos
Lg (mm)	7.16	8.09	6.74	5.67	8.01
Wg	4.57	5.12	4.16	3.60	5.51
Wo	3.58	3.64	3.52	2.63	3.95
Wc	14.64	15.12	16.53	11.95	17.70
Lp	4.20	4.32	3.77	2.45	4.25
Wp	10.50	10.80	10.21	6.77	10.85
TL	17.41	18.70	16.70	13.02	18.26
Lg/Wc	0.49	0.53	0.41	0.47	0.45
D (mm)	0	41.42	36.34	65.21	36.36
D/meanTL		2.46	2.16	3.88	2.16
α (°)	0	0.96	14.09	11.14	14.65

mean TL (mm)	16.82
mean α (°)	13.97
mean D (mm)	44.83

LC09 (BOM 1704) N=3

	1	2	3
pos/neg	pos	pos	pos
Lg (mm)	9.22	9.60	7.32
Wg	6.44	6.18	5.01
Wo	4.25	nd	4.19
Wc	18.44	20.11	17.61
Lp	4.474	4.36	3.61
Wp	12.68	13.20	11.30
TL	21.14	22.60	19.38
Lg/Wc	0.5	0.48	0.42
D (mm)	0	30.69	31.58
D/meanTL		1.46	1.50
α (°)	0	32.94	48.57

mean TL (mm)	21.04
mean α (°)	40.75
mean D (mm)	31.14

LC15 (AA.TER.OI.13) N=16

	1	2	3	4	5	6	7	8	9	10	11	12	13	14	15	16
pos/neg	pos	pos	pos	pos	pos	pos	pos	pos	pos	pos	pos	pos	pos	pos	pos	pos
Lg (mm)	nd	nd	nd	nd	9.81	nd	7.77	7.05	5.59	9.41	nd	7.61	5.92	6.87	6.16	nd
Wg	nd	nd	nd	nd	6.75	nd	5.39	5.51	4.55	6.26	5.56	4.55	4.08	4.29	3.82	nd
Wo	nd	nd	nd	nd	nd	nd	5.54	5.01	nd	5.74	5.42	3.93	2.85	3.66	nd	nd
Wc	nd	nd	nd	7.195	22.38	14.58	16.74	19.20	nd	nd	21.89	17.58	13.17	13.30	15.06	nd
Lp	nd	nd	nd	nd	5.94	4.97	5.19	5.10	nd	nd	5.96	4.52	3.07	3.74	nd	nd
Wp	nd	nd	nd	nd	13.89	11.98	12.42	11.76	nd	nd	12.56	9.83	8.84	10.47	nd	nd
TL	nd	20.70	nd	nd	23.79	nd	20.40	19.90	nd	nd	22.47	16.40	14.12	15.02	nd	nd
Lg/Wc	nd	nd	nd	nd	0.44	nd	0.46	0.37	nd	nd	nd	nd	0.45	0.52	0.41	nd
D (mm)	0	70.72	13.06	7.57	28.17	14.66	12.12	41.75	26.57	15.58	14.40	89.97	26.47	19.93	13.09	17.61
D/meanTL	nd	nd	nd	nd	nd	nd	nd	nd	nd	nd	nd	nd	nd	nd	nd	nd
α (°)	0	29.92	6.28	66.74	68.50	1.88	0.03	33.22	128.79	152.33	18.43	2.90	83.48	118.00	43.00	nd

mean TL (mm)	19,1
mean α (°)	53.82
mean D (mm)	27.44

LC17 (MGL 096718) N=5

	1	2	3	4	5
pos/neg	pos	pos	pos	pos	pos
Lg (mm)	7.78	6.54	nd	8.08	7.14
Wg	5.59	5.22	nd	nd	5.57
Wo	5.13	4.33	nd	nd	nd
Wc	18.61	16.41	17.80	17.64	17.81
Lp	5.41	4.044	4.50	5.21	4.40
Wp	12.48	10.34	12.11	12.75	11.95
TL	19.37	16.80	nd	20.65	17.46
Lg/Wc	0.42	0.40	nd	0.46	0.40
D (mm)	0	23.30	9.38	23.09	21.71
D/meanTL	0	1.25	0.50	1.24	1.17
α (°)	0	10.61	0.66	38.73	11.33

mean TL (mm)	18.57
mean α (°)	15.33
mean D (mm)	19.37

SUPPLEMENTARY TABLE 3 (part 2/2)

LC18 (MGL 096727) N=22

	1	2	3	4	5	6	7	8	9	10	11	12	13	14	15	16	17	18	19	20	21	22
**pos/neg**	pos	pos	pos	pos	pos	pos	pos	pos	pos	pos	pos	pos	pos	pos	pos	pos	pos	pos	pos	pos	pos	pos
**Lg** (mm)	6.30	6.79	8.01	8.13	nd	6.78	6.83	6.94	nd	nd	nd	6.28	7.63	nd	nd	7.81	6.51	nd	8.53	nd	nd	nd
**Wg**	4.68	5.37	6.07	6.65	nd	5.20	5.43	4.99	nd	nd	nd	4.73	5.07	nd	nd	nd	4.78	nd	6.39	nd	nd	nd
**Wo**	nd	4.79	4.71	4.5	nd	nd	4.55	4.57	nd	nd	nd	nd	4.51	nd	nd	4.34	5.04	nd	5.04	nd	nd	5.00
**Wc**	17.56	17.15	20.63	17.20	nd	15.82	15.99	17.08	nd	16.86	nd	nd	16.30	nd	nd	17.43	17.00	nd	17.75	nd	nd	18.40
**Lp**	5.80	4.28	4.17	4.94	4.37	3.73	3.92	4.482	nd	nd	nd	nd	4.55	3.71	nd	4.78	3.93	nd	4.23	nd	nd	4.29
**Wp**	13.00	10.60	12.34	10.53	11.15	9.77	11.33	11.16	nd	nd	nd	nd	11.71	10.07	12.90	11.43	10.47	nd	12.62	nd	nd	10.84
**TL**	17.39	17.94	20.60	20.44	nd	15.58	16.830	18.04	17.65	nd	19.87	nd	18.97	nd	nd	19.98	16.42	nd	18.62	nd	nd	17.58
**Lg/Wc**	0.36	0.39	0.39	0.47	nd	0.43	0.43	0.40	nd	nd	nd	nd	0.47	nd	nd	0.45	0.38	nd	0.48	nd	nd	nd
**D** (mm)	0	22.31	15.54	42.99	7.65	19.98	22.78	20.01	13.12	18.14	6.56	41.21	10.26	18.65	2.50	19.28	46.09	6.69	17.23	28.99	3.83	11.88
**D/meanTL**		1.22	0.85	2.35	0.42	1.09	1.24	1.09	0.72	0.99	0.36	2.25	0.56	1.02	0.13	1.05	2.52	0.38	0.94	1.58	0.21	0.65
**α** (°)	0	43.53	39.43	2.09	1.47	14.37	7.59	2.51	5.74	5.84	13.06	18.68	4.34	21.15	11.34	9.23	10.34	5.69	17.07	9.61	60.73	60.21

**mean TL** (mm)	**18.28**
**mean α** (°)	**17.33**
**mean D** (mm)	**18.84**

LC19 (MGL 097761) N=6

	1	2	3	4	5	6
**pos/neg**	pos	pos	pos	pos	pos	pos
**Lg** (mm)	6.90	7.25	nd	7.56	7.45	7.55
**Wg**	nd	5.05	nd	5.57	5.69	5.37
**Wo**	nd	5.25	nd	nd	nd	5.70
**Wc**	19.56	16.88	nd	15.41	17.19	21.20
**Lp**	nd	4.408	nd	nd	4.520	4.89
**Wp**	nd	12.8	nd	nd	13.01	12.81
**TL**	nd	18.94	nd	nd	20.18	20.16
**Lg/Wc**	0.35	0.43	nd	0.49	0.43	0.36
**D** (mm)	0	12.26	12.94	29.83	9.95	16.29
**D/meanTL**	0	0.62	0.65	1.51	0.50	0.82
**α** (°)	0	12.01	3.62	28.68	6.49	16.54

**mean TL** (mm)	**19.76**
**mean α** (°)	**13.47**
**mean D** (mm)	**16.25**

LC20 (MHNM 15690-185) N=5

	1	2	3	4	5
**pos/neg**	pos	pos	pos	pos	pos
**Lg** (mm)	7.00	7.90	8.87	nd	6.88
**Wg**	6.24	6.10	5.94	nd	5.78
**Wo**	4.35	5.20	4.90	nd	4.52
**Wc**	17.21	16.17	17.15	17.15	15.82
**Lp**	3.75	5.06	4.94	3.48	3.97
**Wp**	11.25	11.82	11.86	10.51	11.17
**TL**	19.62	21.42	20.90	nd	17.60
**Lg/Wc**	0.41	0.49	0.52	nd	0.43
**D** (mm)	0	28.91	48.7	13.27	11.84
**D/meanTL**	0	1.45	1.05	0.68	0.59
**α** (°)	0	3.23	8.38	0.25	13.79

**mean TL** (mm)	**19.89**
**mean α** (°)	**6.41**
**mean D** (mm)	**25.68**

LC21 (AA.TER.OI.12) N=11

	1	2	3	4	5	6	7	8	9	10	11
**pos/neg**	pos	pos	pos	pos	pos	pos	pos	pos	pos	pos	pos
**Lg** (mm)	5.22	8.71	7.92	7.74	8.63	nd	8.46	4.90	nd	8.50	nd
**Wg**	3.52	5.83	5.65	5.36	5.44	nd	4.58	nd	nd	5.26	nd
**Wo**	nd	5.36	4.77	nd	nd	nd	nd	nd	nd	4.75	nd
**Wc**	15.07	19.49	18.00	19.56	19.34	nd	19.58	14.08	9.83	17.94	14.08
**Lp**	3.26	4.271	nd	nd	4.04	nd	3.73	2.86	1.89	3.96	2.50
**Wp**	9.38	12.42	nd	nd	13.38	nd	11.34	8.54	6.76	11.77	10.48
**TL**	14.71	21.12	19.68	nd	19.64	nd	18.17	12.22	8.60	18.08	nd
**Lg/Wc**	0.35	0.45	0.44	0.39	0.45	nd	0.43	0.35	nd	0.47	nd
**D** (mm)	0	32.82	31.86	22.63	11.29	9.27	16.06	46.31	6.39	71.84	25.63
**D/meanTL**	0	1.98	1.93	1.37	0.68	0.56	0.97	2.8	0.39	4.35	1.55
**α** (°)	0	15.43	0.24	3.14	0.41	14.05	14.06	4.90	4.53	13.15	4.5

**mean TL** (mm)	**16.53**
**mean α** (°)	**7.44**
**mean D** (mm)	**27.41**

α = inter-individual angle
D = inter-individual distance
LC = linear cluster (coll. nb in brackets)
Lg = glabella length
Lp = pygidium length
nd = no data
pos/neg = positive/negative relief
TL = total length
Wc = cranidium width
Wg = glabella width
Wo = occipital ring width
Wp = pygidium width
max/min
1 to X= trilobite (*Ampyx priscus*) number

clockwise | anticlockwise
