# Supplementary material for: Collective behaviour in 480-million-year-old trilobite arthropods from Morocco
Source: Sci Rep. 2019 Oct 17;9:14941. doi: 10.1038/s41598-019-51012-3 (PMC6797724; doi:10.1038/s41598-019-51012-3)
Supplement: Supplementary file 3 — SUPPLEMENTARY FIGURES 1-13 [file 41598_2019_51012_MOESM3_ESM.pdf]

Collective behaviour in 480-million-year-old trilobite arthropods from Morocco

Jean Vannier, Muriel Vidal, Robin Marchant, Khadija El Hariri, Khaoula Kouraiss, Bernard Pittet, Abderrazak El Albani, Arnaud Mazurier, and Emmanuel Martin

SUPPLEMENTARY FIGURES 1-13

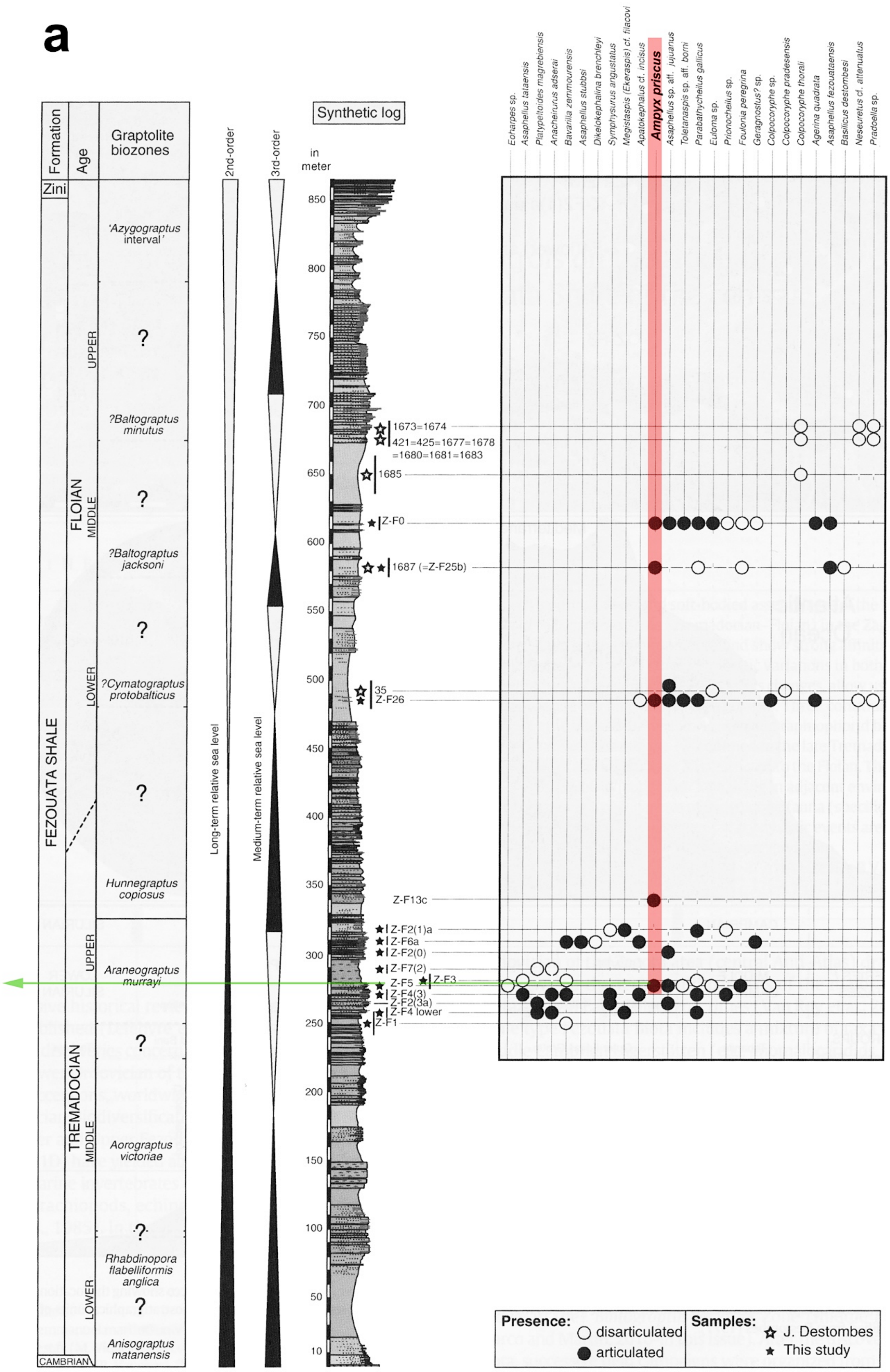
a
Formation
Age
Graptolite biozones
2nd-order
3rd-order
Synthetic log
in meter
Zini
FEZOUATA SHALE
CAMBRIAN
FLOIAN
TREMADOCIAN
UPPER
MIDDLE
LOWER
'Azygograptus interval'
?Baltograptus minutus
?Baltograptus jacksoni
?Cymatograptus protobalticus
Hunnegraptus copiosus
Araneograptus murrayi
Aorograptus victoriae
Rhabdinopora flabelliformis anglica
Anisograptus matanensis
Long-term relative sea level
Medium-term relative sea level
1673=1674
421=425=1677=1678
=1680=1681=1683
1685
Z-F0
1687 (=Z-F25b)
35
Z-F26
Z-F13c
Z-F2(1)a
Z-F6a
Z-F2(0)
Z-F7(2)
Z-F5
Z-F3
Z-F4(3)
Z-F2(3a)
Z-F4 lower
Z-F1
Eoharpes sp.
Asaphellus tataensis
Platypeltoides magrebiensis
Anacheirurus adserai
Bavarilla zemmourensis
Asaphellus stubbsi
Dikelokephalina brenchleyi
Symphysurus angustatus
Megistaspis (Ekeraspis) cf. filacovi
Apatokephalus cf. incisus
Ampyx priscus
Asaphellus sp. aff. jujuanus
Toletanaspis sp. aff. borni
Parabathycheilus gallicus
Euloma sp.
Prionocheilus sp.
Foulonia peregrina
Geragnostus? sp.
Colpocoryphe sp.
Colpocoryphe pradesensis
Colpocoryphe thorali
Agerina quadrata
Asaphellus fezouataensis
Basilicus destombesi
Neseuretus cf. attenuatus
Pradoella sp.
Presence:
disarticulated
articulated
Samples:
J. Destombes
This study

b

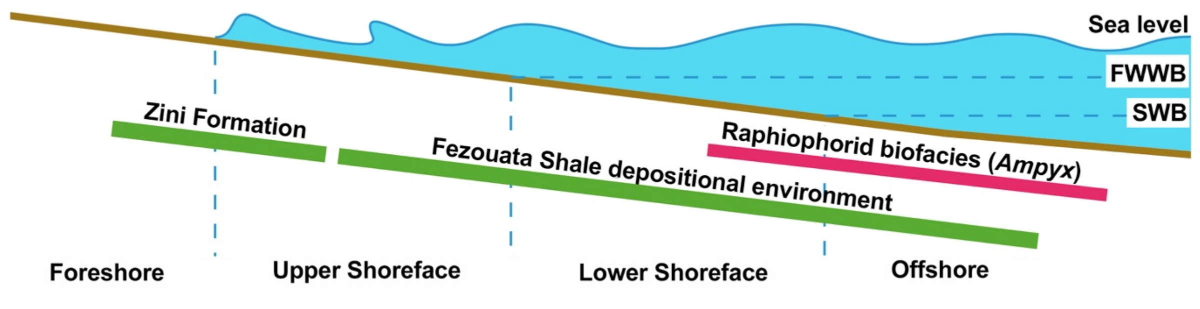

c

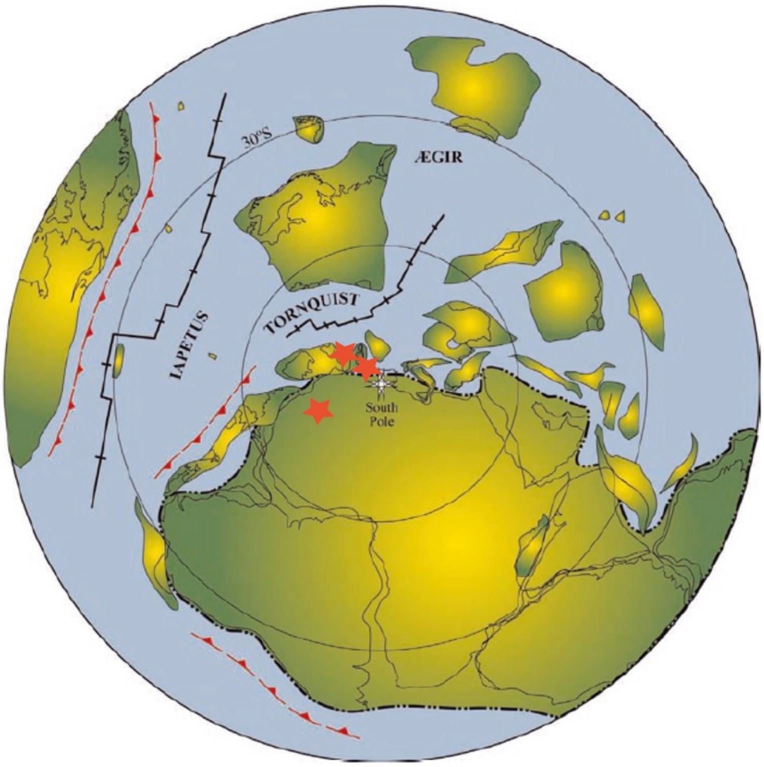

Lower Ordovician (ca 480 Ma)

Supplementary Figure 1| *Ampyx priscus* from the Lower Ordovician (Upper Tremadocian-Floian) of Morocco (Zagora area). a, Vertical distribution (red line) through the Fezouata Shale and associated fauna; green line indicates lowermost occurrence; modified from Martin et al.[7]. **b**, Palaeobathymetrical profile showing the distribution of lithological formations (green lines) and raphiophorid biofacies to which *Ampyx* belongs (red line); modified from Martin et al.[7,12] and Vaucher et al.[21,22]. **c**, Occurrence of *Ampyx priscus* along the peri-Gondwanan margins (red stars correspond to Morocco, southern part of France and Sardinia (from Cocks & Torsvik[18]). References are given in Supplementary Text.

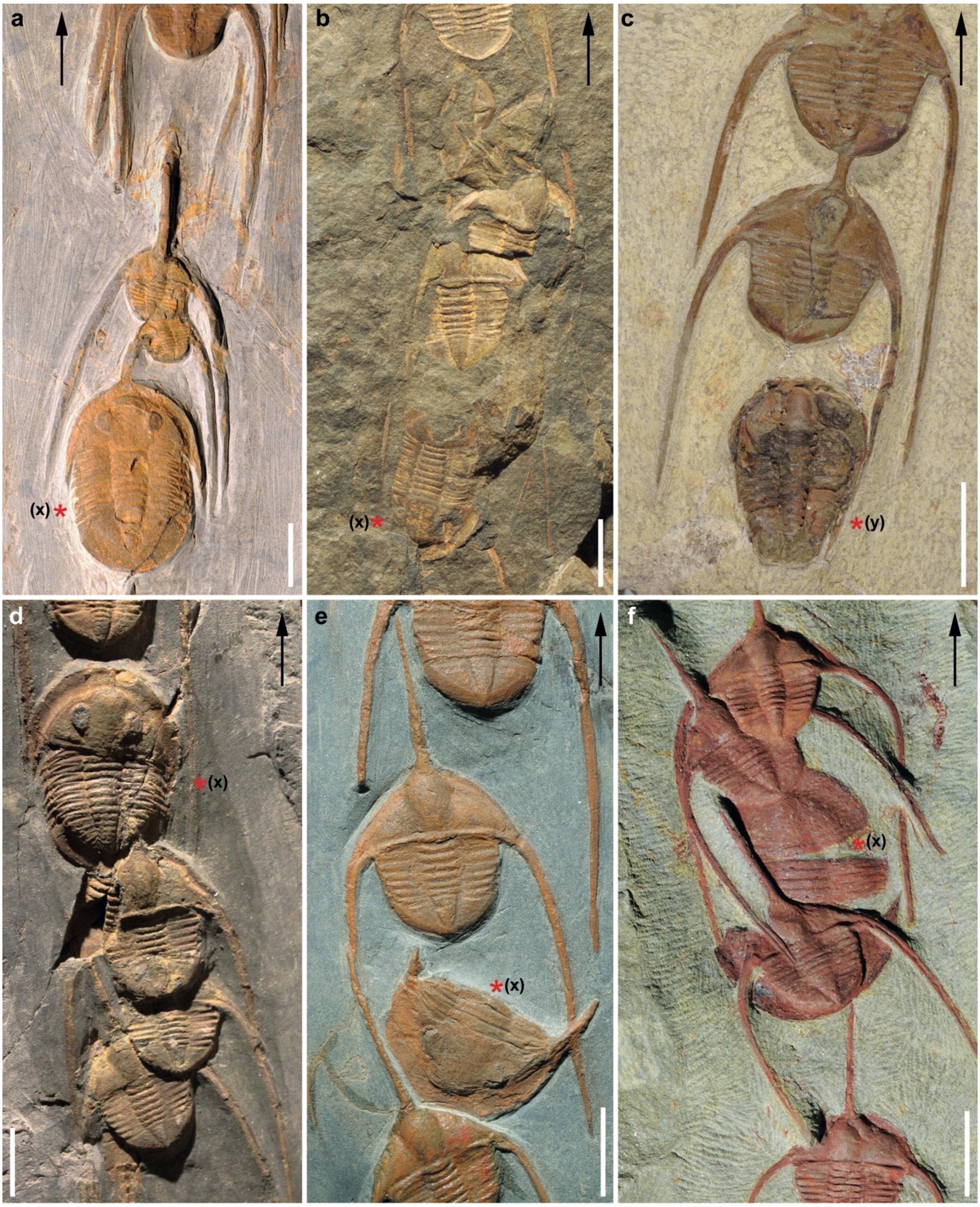

Supplementary Figure 2| Non-*Ampyx* elements in trilobite linear clusters from the Lower Ordovician (Upper Tremadocian-Floian) Fezouata Shale of Morocco (Zagora area), indicated by red asterisks. **a**, AA.TER.OI.12 (see general view in Fig. 1a, b). **b**, AA.TER.OI.13 (see general view in Figs. 1d and Supplementary Fig. 3). **c**, MGL 096718 (see general view in Supplementary Fig. 5b). **d**, MHNM 15690-185 (see general view in Supplementary Fig. 5c) . **e**, BOM 1703 (see general view in Supplementary Fig. 4a*)*. **f**, BOM 2461 (see general view in Fig. 1e). Black arrows indicate prevalent direction of *Ampyx* specimens in each cluster. All light photographs. Abbreviations are as follows: (x), *Asaphellus* aff. *jujuanus* (possibly enrolled in **e;** disarticulated in **f**); (y) *Parabathycheilus* sp. Scale bars: 1 cm.

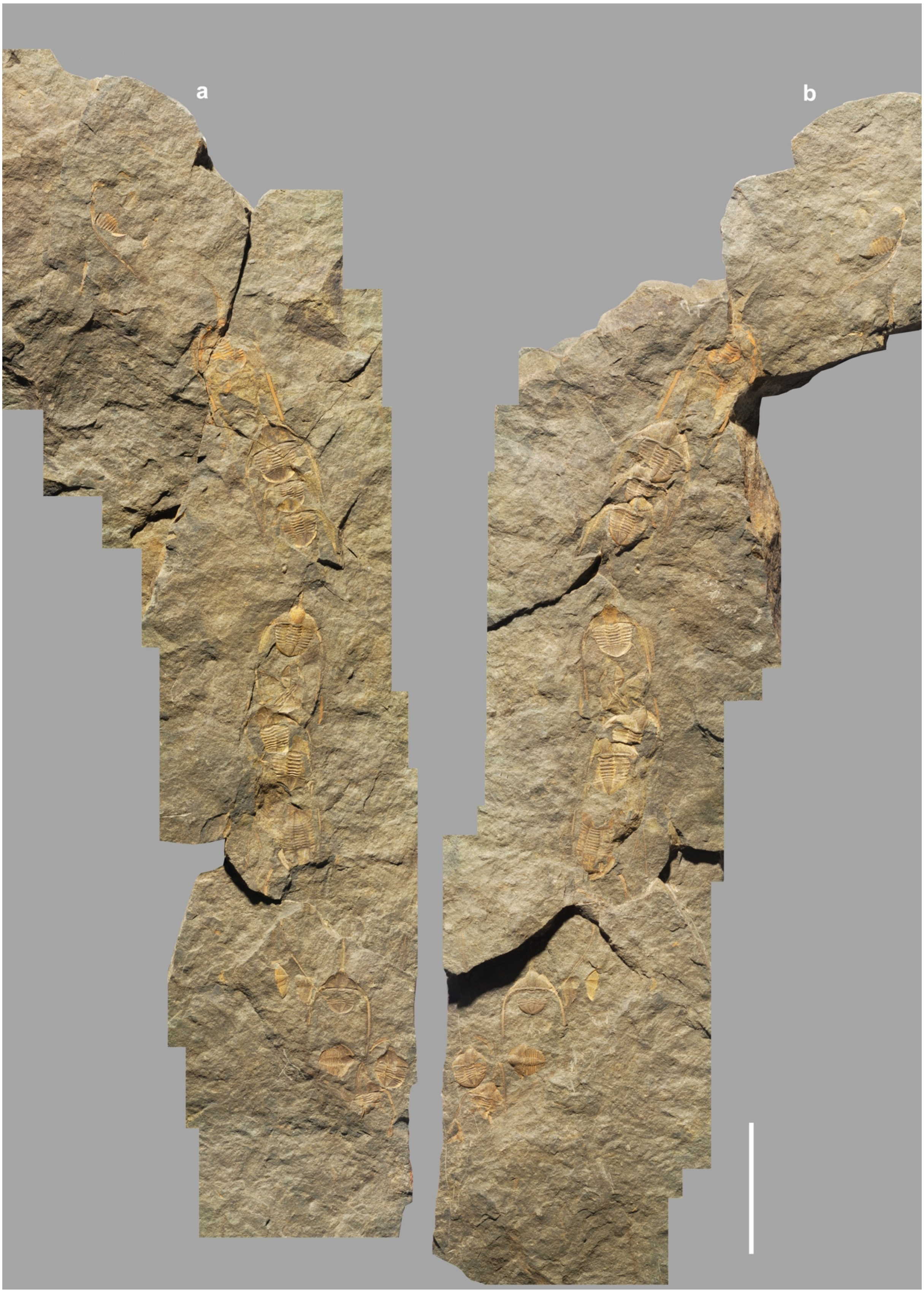

Supplementary Figure 3| ***Ampyx priscus*** linear cluster from the Lower Ordovician (Upper Tremadocian-Floian) Fezouata Shale of Morocco (Zagora area), AA.TER.OI.13 (see Fig. 1d), part and counterpart. Light photographs. Scale bar: 5 cm.

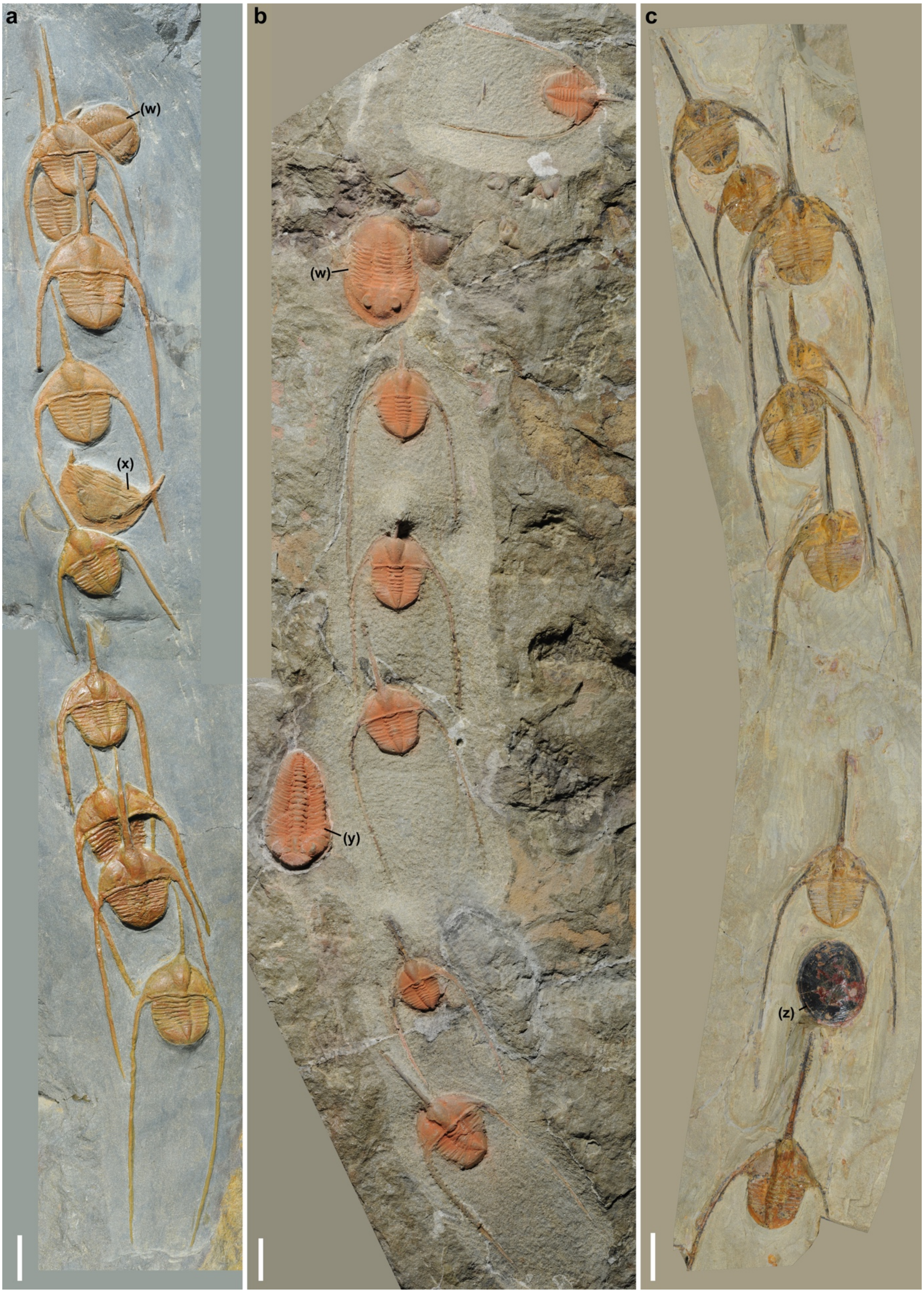

Supplementary Figure 4| ***Ampyx priscus*** linear clusters from the Lower Ordovician (Upper Tremadocian-Floian) Fezouata Shale of Morocco (Zagora area). **a**, BOM 1703. **b**, BOM 1801. **c**, BOM 1235. All light photographs. Abbreviations are as follows: (w), undet. asaphid; (x), *Asaphellus* aff. *jujuanus*; (y), *Toletanaspis* sp.; (z), inarticulate brachiopod. Scale bars: 1 cm.

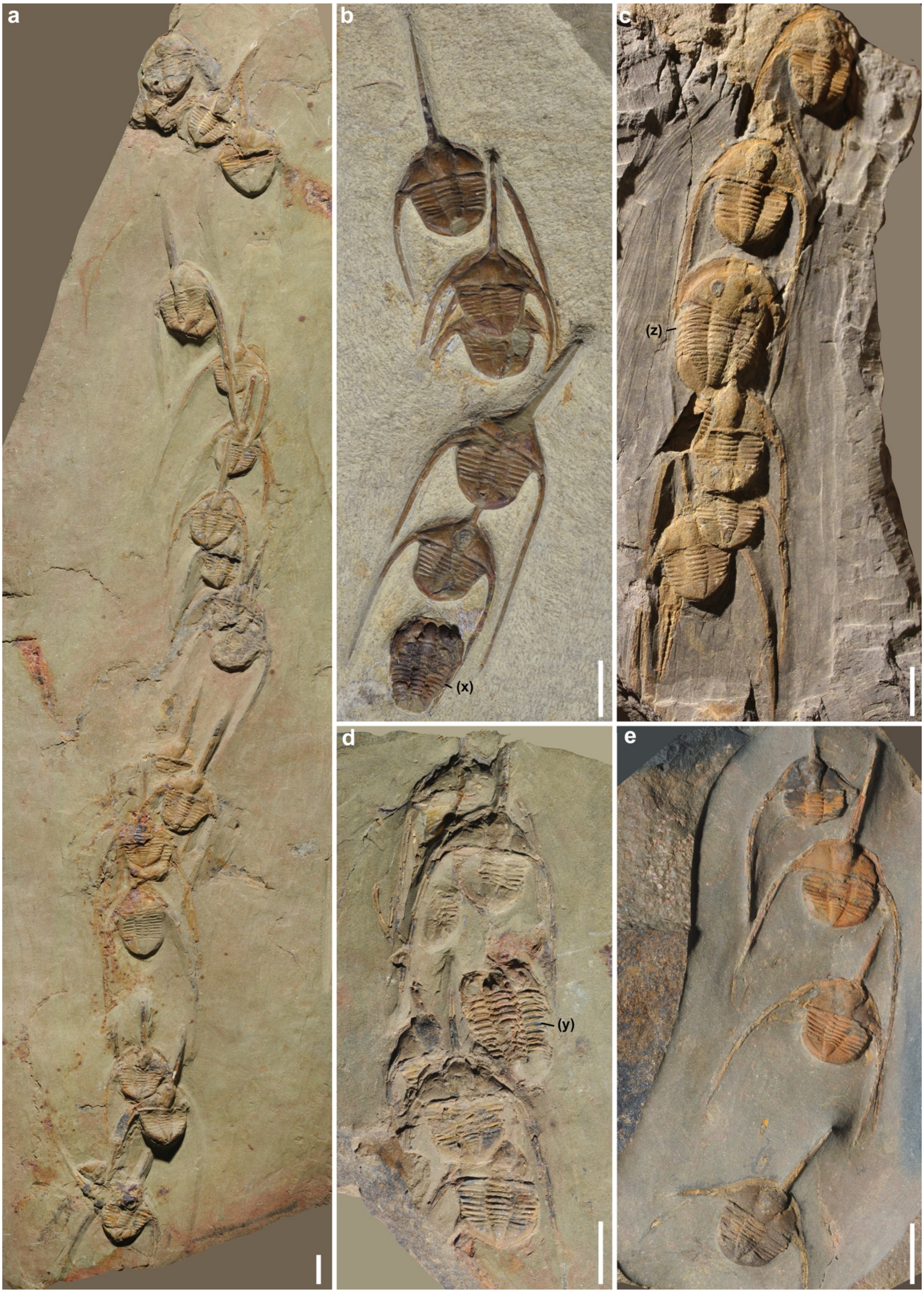

Supplementary Figure 5| ***Ampyx priscus*** linear clusters from the Lower Ordovician (Upper Tremadocian-Floian) Fezouata Shale of Morocco (Zagora area). **a**, MGL 096727 (see Fig. 1c). **b**, MGL 096718. **c**, MHNM 15690-185. **d**, MGL 097761. **e**, BOM 1442. Abbreviations are as follows: (x) and (y), *Parabathycheilus* sp.; (z), *Asaphellus* aff. *jujuanus*. All light photographs. Scale bars: 1 cm

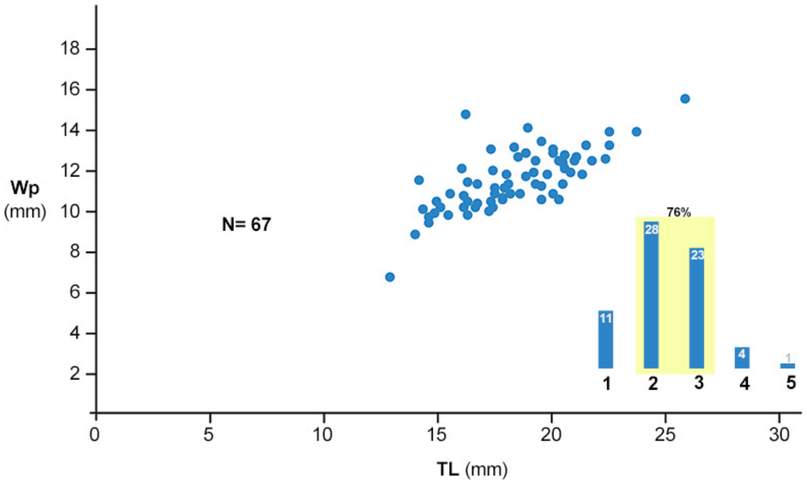

Supplementary Figure 6| Variation of the total length (TL) relative to the width of pygidium (Wp) in 67 specimens of *Ampyx priscus* (linear clusters) from the Lower Ordovician (Upper Tremadocian-Floian) Fezouata Shale of Morocco (Zagora area)**.** Histograms showing the distribution of TL in 5 classes (1: 13 to 15.99 mm; 2: 16 to 18.99; 3: 19 to 21.99; 4: 22 to 24,99; 5: 25 to 27,99). 76% of the specimens are between 16 and 21,99 mm (TL).

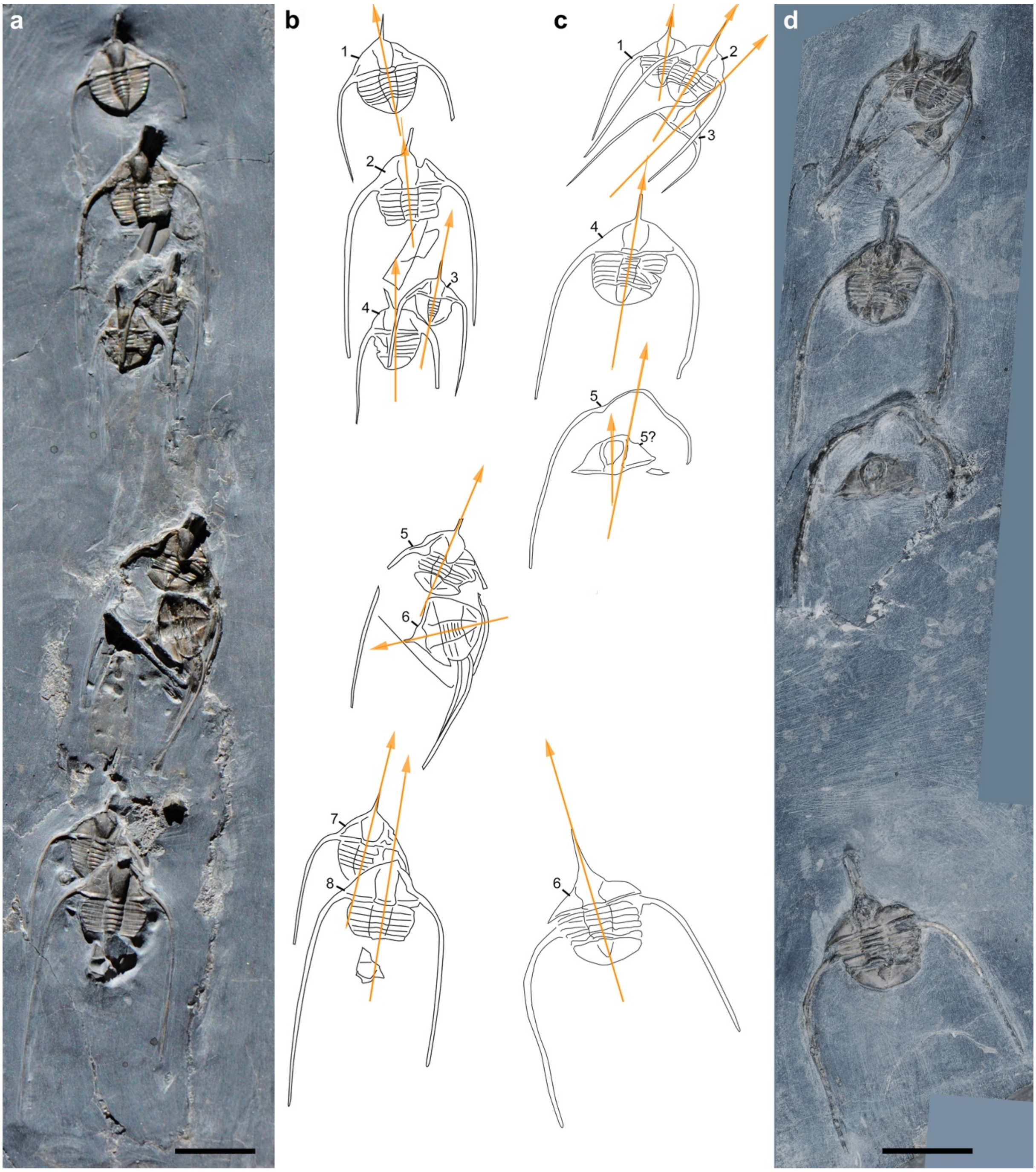

Supplementary Figure 7| ***Ampyx priscus*** linear clusters from the Lower Ordovician (Upper Tremadocian-Floian) Saint-Chinian Formation, Forbidarias Ravine, St. Chinian, Montagne-Noire (Hérault, France). **a, b**, BOM 2480; **c, d**, LAC 001. Scale bars: 1 cm.

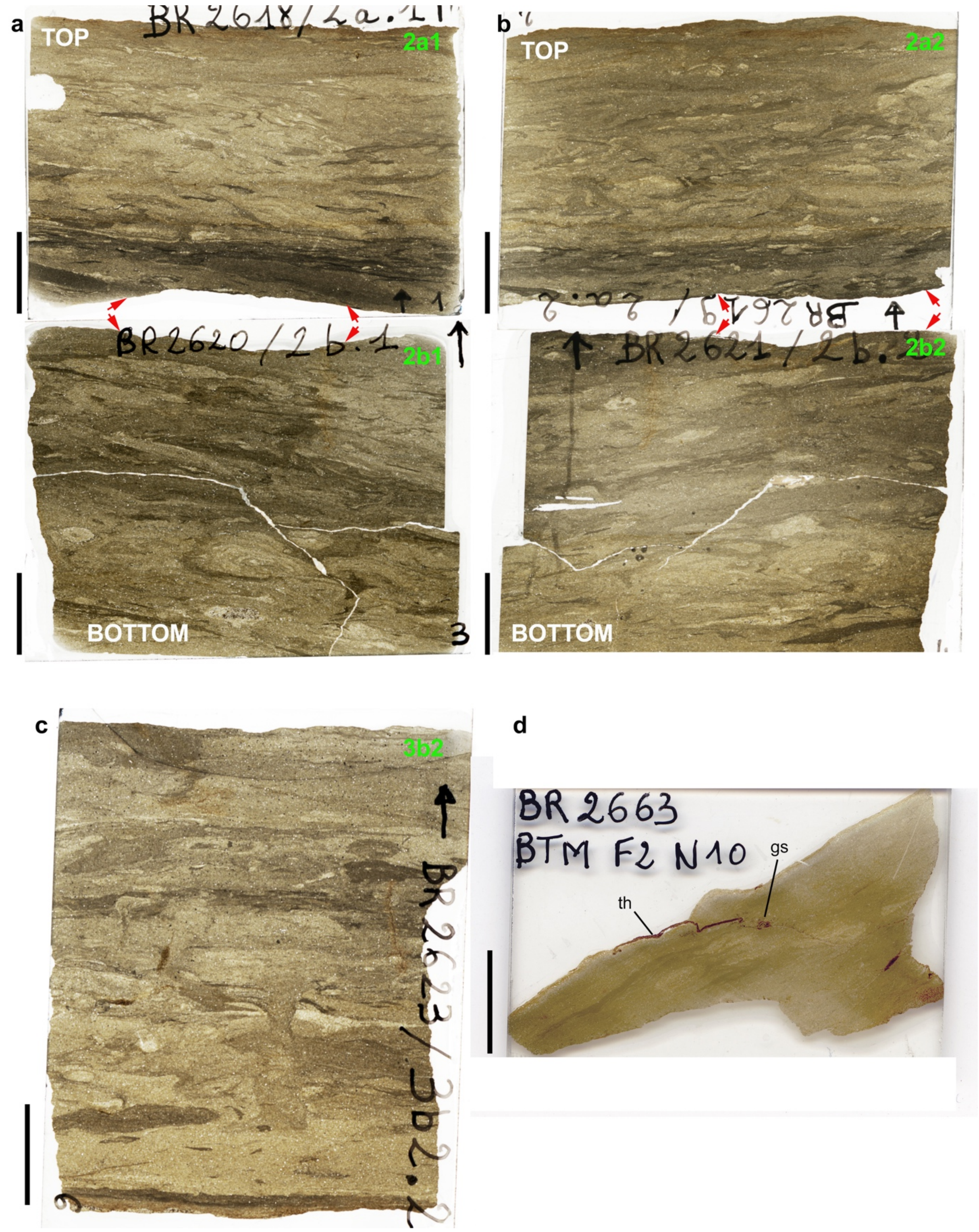

Supplementary Figure 8|a-c, Lithological thin sections through part and counterpart of *Ampyx priscus* linear cluster AA.TER.OI.13 from the Lower Ordovician (Upper Tremadocian-Floian) Fezouata Shale of Morocco (Zagora area). **d,** Thin section through *Ampyx priscus* linear cluster AA.OBZ2.OI.1, also from the Fezouata Shale**.** Red arrows indicate bedding plane with *Ampyx priscus* clusters. Green numbers identify thin section through rock slabs (see location of sections in Fig. 3). Abbreviations are as follows: gs, genal spine; th, thorax. Scale bars: 1 cm.

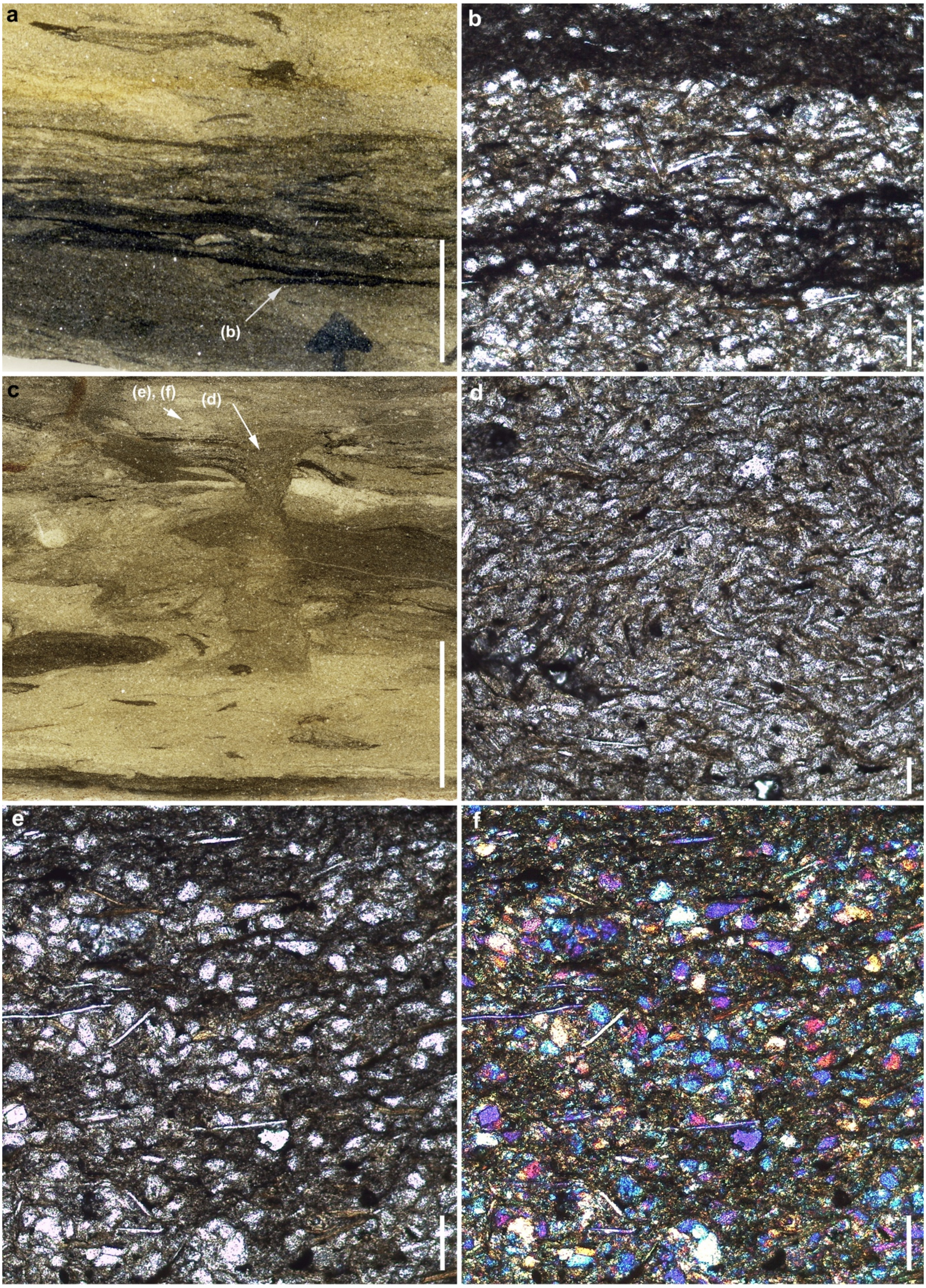

Supplementary Figure 9| Thin sections through rock slab (AA.TER.OI.13) from the Lower Ordovician (Upper Tremadocian-Floian) Fezouata Shale of Morocco (Zagora area). **a, b,** Section 2a1 (see location in Fig. 3 and Supplementary Fig. 8) showing organic-rich layers. **c-f**, Section 3b2-2 (see location in Fig. 3 and Supplementary Fig. 8) showing bioturbation (c, d) and grain size (e, f). Scales bars: 1 cm in c, 5 mm in a, 50 µm in b, d-f.

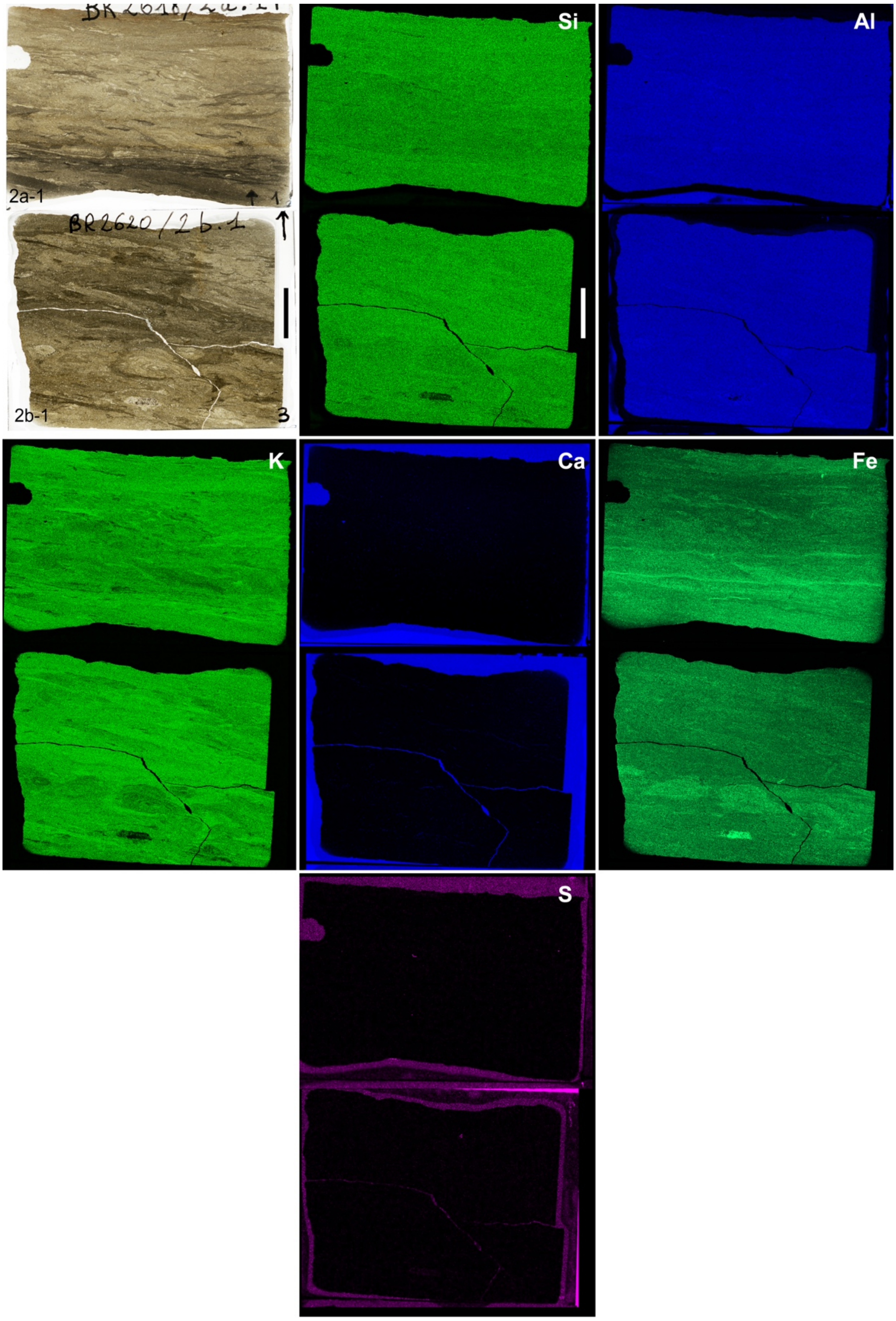

Supplementary Figure 10| Elemental mapping through part and counterpart of rock slab AA.TER.OI.13 (see location in Fig. 3 and thin sections 2a1 and 2b1 in Supplementary Fig. 8), from the Lower Ordovician (Upper Tremadocian-Floian) Fezouata Shale of Morocco (Zagora area). A is photograph of uncovered thin sections. Scale bars: 1 cm.

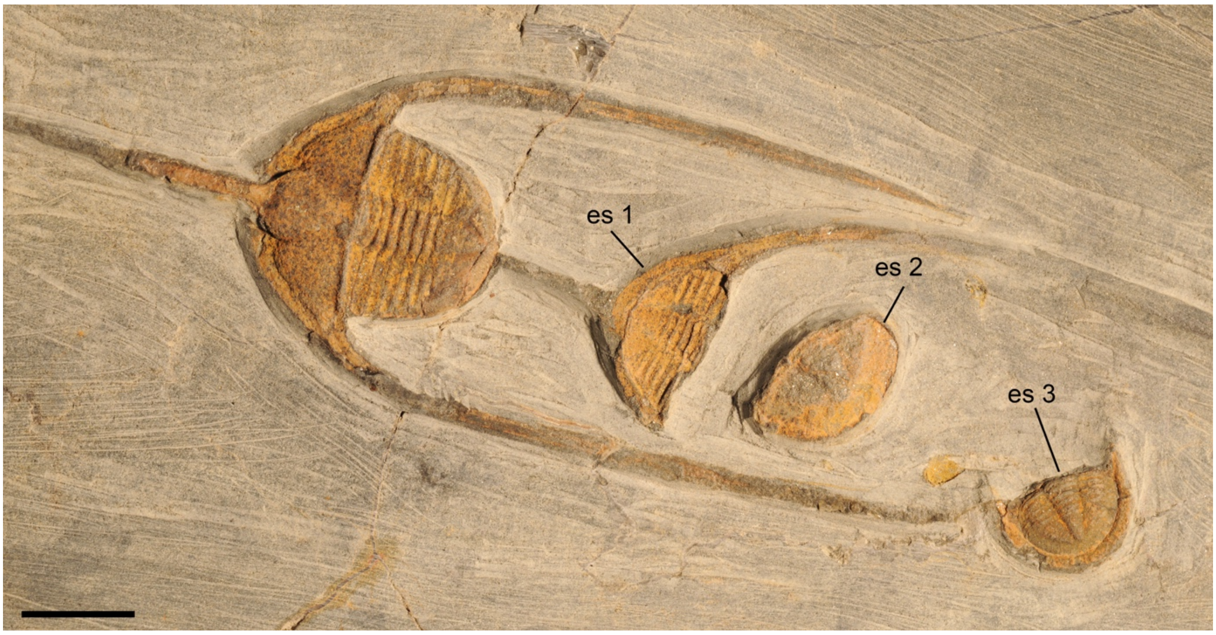

Supplementary Figure 11| Enrollment in *Ampyx priscus* from the Lower Ordovician (Upper Tremadocian-Floian) Fezouata Shale of Morocco (Zagora area). AA.TER.OI.12, posterior part of linear cluster (see Figure 1a for general view) showing one enrolled specimen (es1). es3 is an enrolled, juvenile asaphid trilobite; es2 is a poorly preserved enrolled (possibly asaphid) specimen. Scale bar: 1 cm.

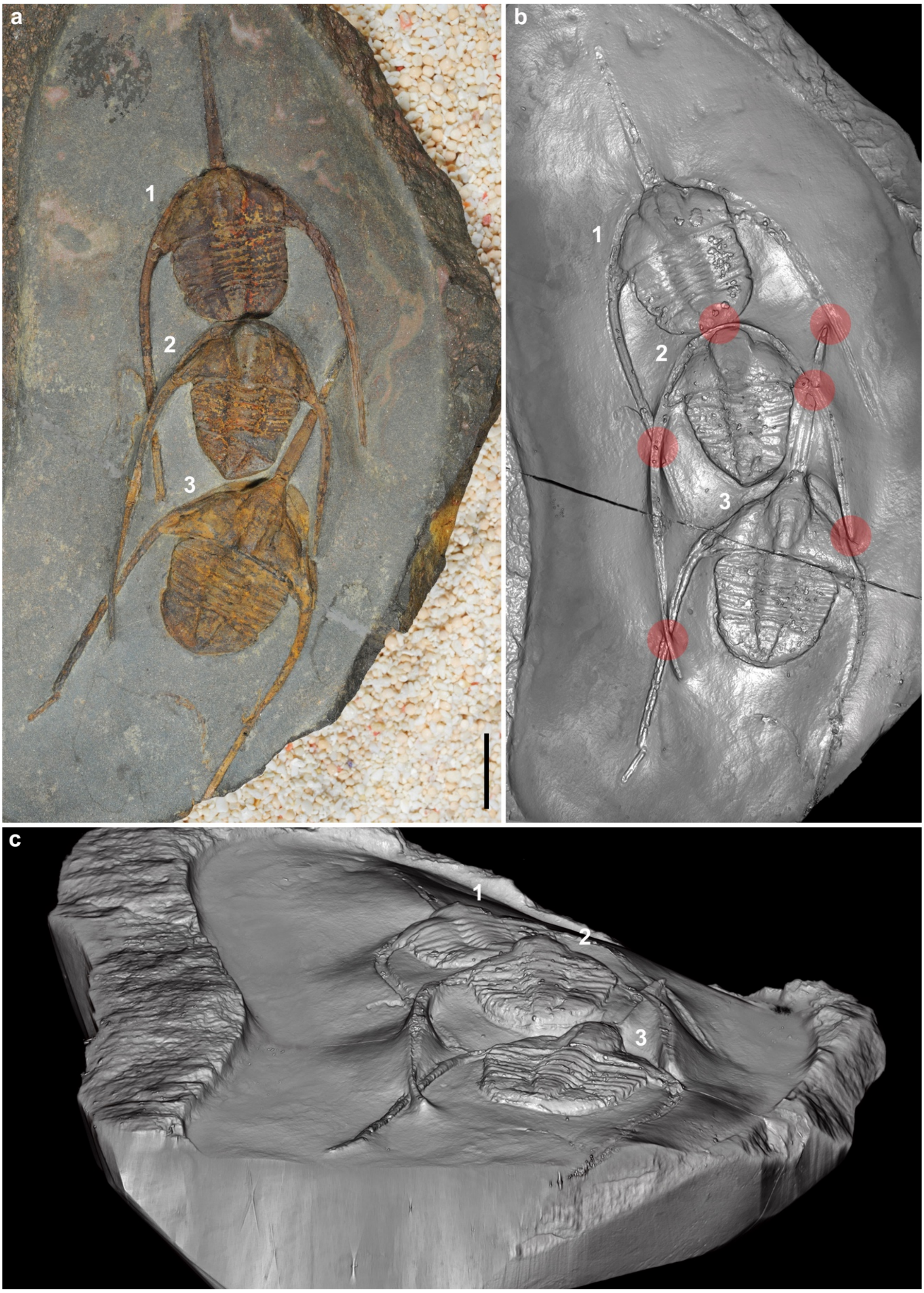

Supplementary Figure 12| ***Ampyx priscus*** linear cluster, ROMIP 57013, from the Lower Ordovician (Upper Tremadocian-Floian) Fezouata Shale of Morocco (Zagora area). **a, b,** lateral view showing contact points (red spots) between three individuals via their genal and glabellar spines and other parts of their exoskeleton. **c**, posterior view of the trilobite cluster. a is a light photograph; b, c are external tomographic images. Scale bar: 1 cm.

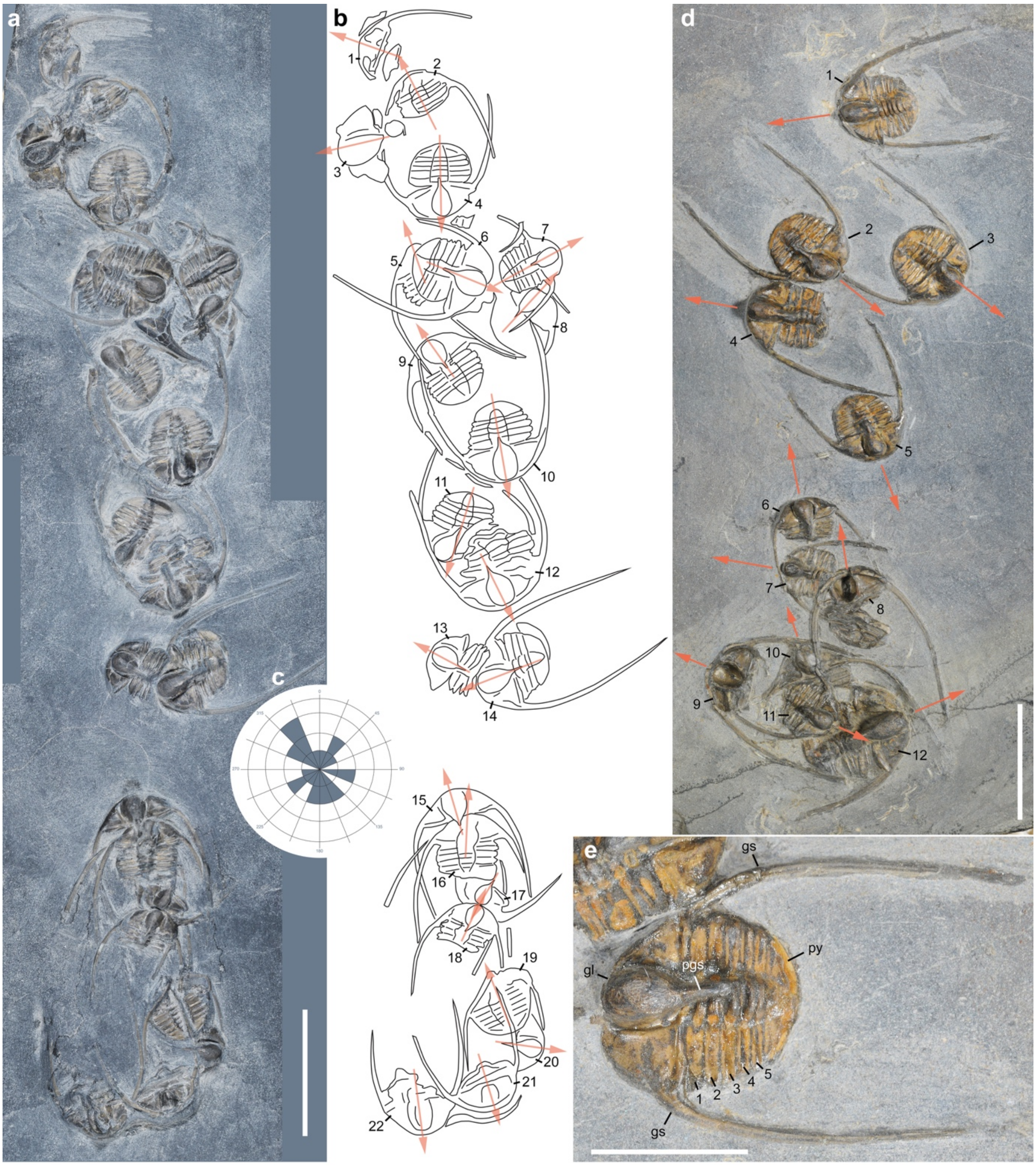

Supplementary Figure 13| ***Ampyxinella (Eoampyxinella) villebruni*** (Thoral, 1935) clusters from the Lower Ordovician (Upper Tremadocian-Floian) Saint-Chinian Formation, Montagne-Noire, Hérault, France. **a-c**, LAC 002, light photograph, line drawing and diagram showing angular variation between longitudinal axis and vertical landmark. **d, e**, LAC 003, general view and details showing main exoskeletal features. Orange arrows indicate longitudinal axis of trilobites and direction. Abbreviations are as follows: gl, glabella; gs, genal spines; pgs, post-glabellar spine; py; pygidium; 1-5, 1st to 5th thoracic segments. Scale bars: 1 cm.
